# Molecular Characterization of Hemopexin in the Siberian Sturgeon (*Acipenser baerii*): Evolutionary Insights and Differential Expression Under Immune and Thermal Stresses

**DOI:** 10.3390/ijms26167934

**Published:** 2025-08-17

**Authors:** Eun Jeong Kim, Ki Hong Kim, Yoon Kwon Nam

**Affiliations:** 1 Faculty of Fisheries Life Sciences, Pukyong National University, Busan 48513, Republic of Korea; kejung03@naver.com; 2 Department of Aquatic Life Medicine, Pukyong National University, Busan 48513, Republic of Korea; khkim@pknu.ac.kr

**Keywords:** *Acipenser baerii*, *HPX-Wap65* evolution, immune response, thermal stress

## Abstract

Hemopexin (HPX) is a heme-binding glycoprotein involved in detoxification, oxidative stress regulation, and immune defense. In teleosts, gene duplication gave rise to *Wap65-1* and *Wap65-2*, but the structure and function of ancestral *HPX* in basal actinopterygians remain poorly understood. Here, we characterized *HPX* from the Siberian sturgeon (*Acipenser baerii*), a slow-evolving chondrostean species that diverged prior to the teleost-specific duplication. Structural modeling and superimposed 3D alignment revealed high similarity between sturgeon HPX, human HPX, and Wap65-2, but not Wap65-1. Phylogenetic analysis placed sturgeon HPX in a distinct basal clade within the Actinopterygian lineage, positioned below the divergence of Wap65 paralogs. Tissue expression was liver-dominant but also evident in skin and kidney, and *HPX* transcripts increased during larval development. Under *Aeromonas hydrophila* challenge, *HPX* was strongly upregulated in immune-relevant tissues. Thermal challenge also induced modest, tissue-specific changes, particularly in peripheral tissues. These results indicate that ancestral *HPX* possessed multivalent stress responsiveness—primarily immune-related—with supplementary thermal sensitivity. The observed functional flexibility is consistent with the hypothesis that ancestral HPX functions were partitioned following *Wap65* gene duplication in teleosts, with *Wap65-1* evolving toward a specialized thermal response role.

## 1. Introduction

Hemopexin (HPX) is a vertebrate-specific glycoprotein that binds free heme with high affinity, playing a crucial role in heme detoxification, iron homeostasis, and inflammation modulation during infection and tissue injury. As a classical acute-phase protein (APP), HPX limits heme-driven oxidative stress and prevents host tissue damage, particularly under hemolytic or septic conditions [1,2,3]. In mammals, *HPX* is predominantly synthesized in the liver and is systemically upregulated during inflammation [1].

In teleost fish, *HPX* orthologs were initially identified as warm-temperature acclimation-associated proteins (*Wap65*), named after a 65 kDa protein upregulated during thermal stress [4,5,6]. Following the teleost-specific third round of whole-genome duplication (3R WGD), two paralogous genes—*Wap65-1* and *Wap65-2*—emerged [7]. Comparative analyses suggest that *Wap65-2* retains high sequence and functional homology with mammalian *HPX*, including conserved heme-binding domains and immune-responsive regulation. In contrast, *Wap65-1* appears to have diverged functionally, contributing more prominently to thermal acclimation and general stress responses [8,9,10]. This gene duplication has served as a model for subfunctionalization and neofunctionalization, raising important questions about the ancestral state, tissue specificity, and regulatory divergence of *HPX*-related genes in vertebrates [7,10].

Among basal actinopterygians (i.e., lineages that diverged before the 3R WGD), sturgeons (Acipenseriformes) are members of the ancient Chondrostei clade, representing an evolutionary lineage that predates the teleost WGD but exhibits independent lineage-specific polyploidy [11,12]. Their unique phylogenetic position offers valuable insight into the pre-duplication architecture and ancestral functions of *HPX*-like genes [13,14]. Additionally, given their endangered status and growing importance in aquaculture for caviar production, understanding immune- and stress-responsive molecules such as *HPX/Wap65* is not only of evolutionary interest but also of practical relevance to sustainable farming practices [15,16,17].

The Siberian sturgeon (*Acipenser baerii*), belonging to the ancient family Acipenseridae, is a key species in both conservation and global aquaculture. Native populations are distributed across the rivers of Siberia from the Ob to the Kolyma and occur in both semi-anadromous and strictly freshwater forms, with Lake Baikal harboring an isolated lacustrine population [18]. Historically, annual commercial catches exceeded 1700 metric tons, but overfishing and other anthropogenic pressures have driven severe population declines [19,20,21]. To counteract these losses, large-scale aquaculture was initiated in the 1970s and is now practiced in multiple countries, including Russia, France, Germany, Italy, Austria, Japan, and Korea, making *A. baerii* one of the principal sturgeon species in the global farming industry [22,23,24]. From a phylogenetic perspective, sturgeons diverged prior to the teleost radiation and thus occupy a basal position within Actinopterygii. Their genomes, including *A. baerii* are not expected to contain both *Wap65-1* and *Wap65-2* paralogs, but instead likely retain a single ancestral *HPX* gene. Despite this, molecular studies on *HPX* in sturgeons remain scarce, with only limited work focusing on sequence identity or immune challenge-related expression [25,26]. Crucially, the broader functional roles of *HPX*—such as thermal responsiveness or tissue-specific transcriptional regulation—have yet to be fully characterized in this lineage.

To address these gaps, the present study aimed to (1) characterize the structural and phylogenetic relationships of Siberian sturgeon (*A. baerii*) *HPX* to vertebrate *HPX* orthologs and teleost *Wap65* paralogs, including three-dimensional protein modeling; and (2) investigate both basal expression patterns (tissue distribution and ontogenetic stage) and differential expression in response to bacterial infection and thermal challenge.

## 2. Results

### 2.1. Characterization of Acipenser baerii HPX cDNA and Deduced Amino Acid Sequences

The cloned cDNA sequence of *Acipenser baerii HPX* was 1800 bp in length, excluding the poly(A+) tail. It consisted of a 5′ untranslated region (5′-UTR) of 23 bp, an open reading frame (ORF) of 1368 bp including the *TAG* stop codon, and a 3′ untranslated region (3′-UTR) of 399 bp excluding the stop codon and poly(A+) tail. A canonical polyadenylation signal (*AATAAA*) was identified 21 bp upstream of the poly(A+) tail. The ORF encodes a polypeptide of 455 amino acids. The theoretical molecular weight of the deduced protein was calculated to be 57.8 kDa, and the predicted isoelectric point (pI) was 6.03.

**Figure 1:**
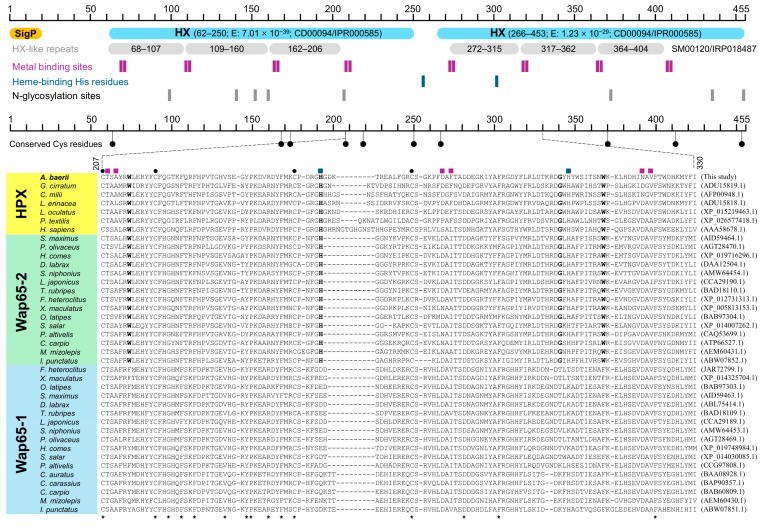
Schematic representation of the conserved domains and functional motifs in the deduced amino acid sequence of *A. baerii* HPX (**top**). The diagram highlights the predicted signal peptide (SigP), two conserved hemopexin (HX) domains, six HX-like repeats, eight putative metal-binding sites, two conserved heme-binding histidine residues, and eight predicted N-glycosylation sites. A partial multiple sequence alignment (**bottom**) with representative HPX, Wap65-1, and Wap65-2 orthologs from vertebrates illustrates the relative conservation of key motifs across species. Five disulfide-bond-forming cysteine pairs are also annotated. *A. baerii* HPX showing higher similarity to Wap65-2 than to Wap65-1. In the alignment, residues conserved for all the species aligned are indicated with asterisks at bottom, while residues conserved only between HPX and Wap65-2 are bolded. Black dots indicate conserved cysteine residues forming disulfide bonds; colored squares denote key motif positions as in the top schematic. The exact positions of residues and/or motifs can be referred to main text.

A signal peptide comprising 19 amino acids was predicted by SignalP 6.0, with a cleavage site between Ala^19^ and Ala^20^. Conserved domain searches using InterPro and CDD identified two hemopexin (HX) domains spanning residues 65–250 and 266–453, respectively. Six HX-like repeat regions were detected (Figure 1). Eight residues involved in metal binding were identified: in the N-domain, D^69^ and I^71^ (DAI), D^110^ and A^112^ (DAA), D^163^ and A^165^ (DAA), and T^208^ and A^210^ (TSA); in the C-domain, D^273^ and F^275^ (DAF), N^318^ and V^320^ (NAV), D^365^ and A^367^ (DAA), and D^408^ and A^410^ (DAA). Two conserved histidine residues related to heme binding were located at His^255^ and His^302^. Five cysteine pairs were predicted to form disulfide bonds, and eight potential N-glycosylation sites were predicted at N^99^, N^140^, N^152^, N^160^, N^206^, N^372^, N^445^, and N^455^ with moderate scores (0.525–0.7288) (Figure 1).

Multiple sequence alignment showed that *A. baerii* HPX shares greater similarity with Wap65-2 than with Wap65-1. Several residues were conserved exclusively between HPX and Wap65-2 but not Wap65-1. The heme-binding histidine in the N-domain was uniquely conserved in HPX/Wap65-2, while the corresponding histidine in the C-domain was present in both Wap65-2 and several Wap65-1 orthologs (Figure 1). Pairwise comparison of amino acid sequence identity and similarity revealed the highest similarity with *Acipenser ruthenus* (98.0% identity, 98.5% similarity), followed by *Lepisosteus oculatus* (55.4% identity, 65.4% similarity). The average identity and similarity with tetrapod HPX orthologs were 37.2 ± 2.7% and 50.8 ± 3.5%, respectively. Comparisons with Wap65-1 showed 41.4 ± 2.1% identity and 56.9 ± 2.1% similarity, while those with Wap65-2 yielded 48.9 ± 1.6% identity and 63.5 ± 1.7% similarity. The identity and similarity scores with Wap65-2 were significantly higher than those with Wap65-1 (*p* < 0.05). No lineage-specific differences were observed across Ostariophysi, Protacanthopterygii, Stomiatii, or Neoteleostei groups (Supplementary Figure S1).

### 2.2. Predicted 3D Structure of A. baerii HPX and Superimposed Visualization of Conserved Motifs with Human HPX

**Figure 2:**
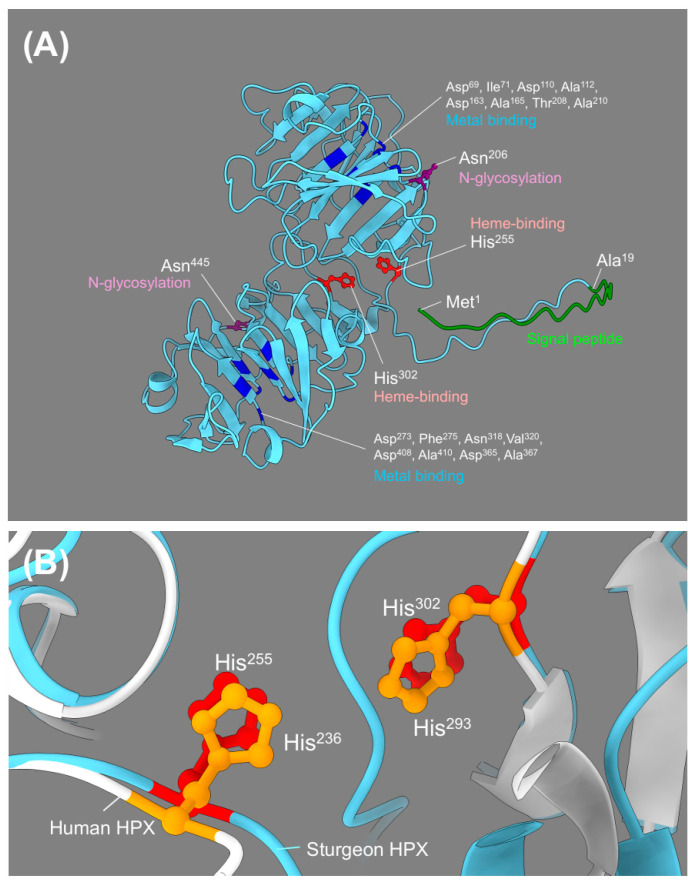
Predicted 3D structure of *A. baerii* HPX and superimposed comparison with human HPX. (**A**) AlphaFold2-predicted tertiary structure of *A. baerii* HPX, revealing a two-domain β-propeller-like architecture characteristic of vertebrate hemopexins. Conserved functional residues are labeled, including signal peptide cleavage site (Ala^19^–Ala^20^), two heme-binding histidines (His^255^ and His^302^), eight metal-binding sites (blue-colored for N- and C-domains), and representative N-glycosylation sites (Asn^206^ and Asn^445^). The quality assessment of modeling can be referred to Supplementary Figure S2. (**B**) Structural superimposition of *A. baerii* and human HPX (P02790) models. Despite low overall sequence identity (33.7%), key heme-binding histidines (human/*A. baerii*; His^236^/His^255^ and His^293^/His^302^) and structurally conserved motifs align well, supporting functional conservation (see Supplementary Figure S3).

The three-dimensional structure of *Acipenser baerii* HPX was predicted using AlphaFold2. The resulting model exhibits a characteristic two-domain β-propeller-like fold, consistent with known hemopexin family structures. An unstructured region was observed immediately following the signal peptide, a feature commonly found in other HPX and Wap65 orthologs. The N- and C-domains are organized in a circular fashion, forming a central cavity, typical of heme-binding hemopexin proteins (Figure 2A; Supplementary Figure S2).

To evaluate structural conservation across species, the *A. baerii* HPX model was superimposed with the crystal structure of human HPX. Despite the relatively low sequence identity between the two proteins (33.7%), the superimposed models showed remarkable similarity in overall architecture. Key functional motifs involved in heme binding, metal coordination, and disulfide bonding were structurally conserved in both proteins (Figure 2B, Supplementary Figure S3).

Superimposed comparison of heme-binding residues revealed that *A. baerii* HPX shares two conserved histidine residues—His^255^ and His^302^—corresponding to His^236^ and His^293^ in human HPX. In contrast, human HPX contains four known histidine residues involved in heme coordination (Supplementary Figure S3A). Residues involved in metal binding were also structurally conserved. Eight key sites (Asp^69^/Ile^71^, Asp^110^/Ala^112^, Asp^163^/Ala^165^, Thr^208^/Ala^210^, Asp^273^/Phe^275^, Asn^318^/Val^320^, Asp^365^/Ala^367^, and Asp^408^/Ala^410^) were all positioned similarly in both models, indicating conserved spatial arrangement (Supplementary Figure S3B). Regarding disulfide bonding, the ten predicted cysteine residues in *A. baerii* HPX showed perfect pairwise matching with those in human HPX. All disulfide bridges appeared conserved in position and orientation, supporting strong structural conservation between the two species (Supplementary Figure S3C). Although both *A. baerii* and human HPX exhibit multiple potential N-glycosylation sites, only two sites—Asn^206^ and Asn^455^ in sturgeon, corresponding to Asn^187^ and Asn^453^ in human HPX—were conserved in both sequence position and spatial localization in the 3D models (Supplementary Figure S3D).

### 2.3. Molecular Phylogenetic Analysis of Vertebrate HPX and Wap65 Orthologs

**Figure 3:**
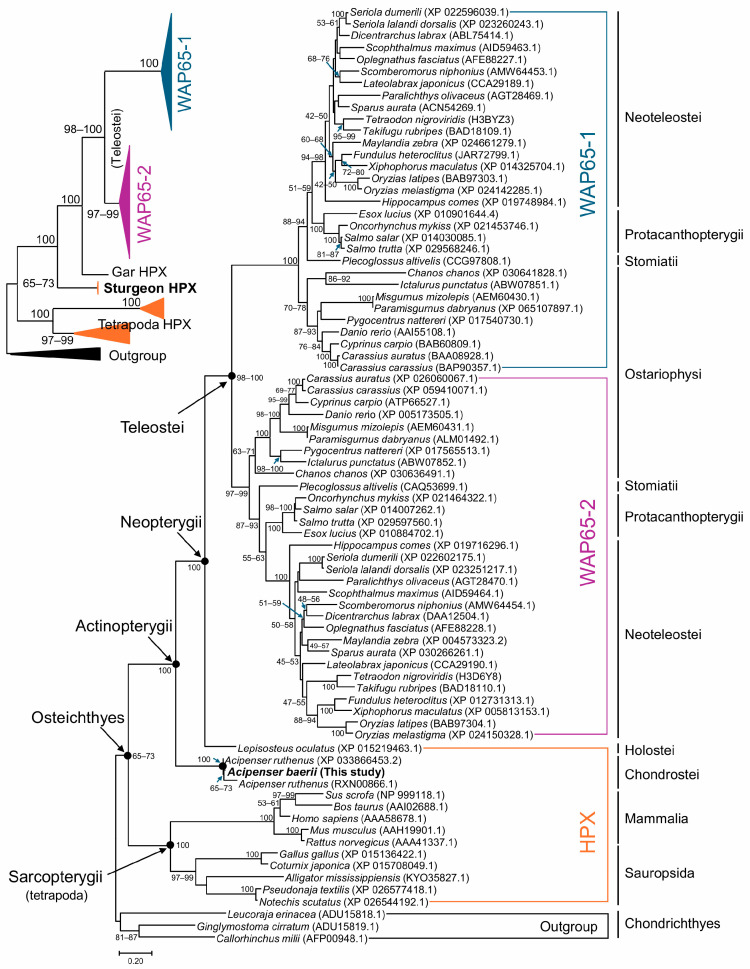
Maximum likelihood tree constructed using amino acid sequences of HPX, Wap65-1, and Wap65-2 orthologs from representative vertebrate species under the JTT model with gamma distribution and invariant sites (G+I), using all alignment sites. The tree is rooted with three cartilaginous fish (Chondrichthyes) sequences used as the outgroup. Support values shown at nodes represent adaptive bootstrap (threshold = 5.0) percentages. A simplified schematic of the major clade topology is provided in the top left panel for reference. *A. baerii* HPX is placed basal to the teleost Wap65-1 and Wap65-2 clades, forming a distinct branch alongside holostean (*Lepisosteus oculatus*) HPX. Phylogenetic trees constructed with a neighbor-joining (NJ) algorithm also displayed the similar tree topologies (see Supplementary Figure S4).

A maximum likelihood (ML) phylogenetic tree was constructed using a dataset of vertebrate HPX and Wap65 amino acid sequences, rooted with three cartilaginous fish HPX sequences as the outgroup. The overall tree topology was consistent with the established taxonomic hierarchy of vertebrates. In the teleost lineage, Wap65 sequences were grouped by isoform (Wap65-1 and Wap65-2) rather than by species, indicating that isoform divergence occurred prior to the radiation of extant teleosts. Within each isoform clade, sequences clustered into subgroups reflecting taxonomic divisions such as Ostariophysi, Stomiatii, Protacanthopterygii, and Neoteleostei, supporting phylogenetic coherence across major teleost groups (Figure 3).

The Wap65 clades formed a large teleostean cluster that was connected to the non-teleost HPX gene of the holostean *Lepisosteus oculatus* (gar), which in turn was connected to the HPX genes of *Acipenser baerii* and *A. ruthenus*, representing the Chondrostei subclass. This positioning reflects the basal placement of Acipenseriformes within the superclass Actinopterygii (ray-finned fishes). The actinopterygian clade was then joined with Sarcopterygii (lobe-finned fishes and tetrapods), forming a monophyletic Osteichthyes (bony fish) group (Figure 3).

To evaluate the robustness of tree topology, additional phylogenetic reconstructions were performed using the neighbor-joining (NJ) method under varying option settings (e.g., substitution models, rate heterogeneity, gap/missing data treatment). Although some minor differences in internal subgrouping were observed among teleost Wap65 isoform clades, the relative placement of *A. baerii* HPX remained consistent—basal within the actinopterygian lineage and distinct from Wap65 isoforms—across all NJ tree topologies (Supplementary Figure S4A,B).

### 2.4. Structural Comparison of A. baerii HPX with Other HPX and Wap65 Orthologs

**Figure 4:**
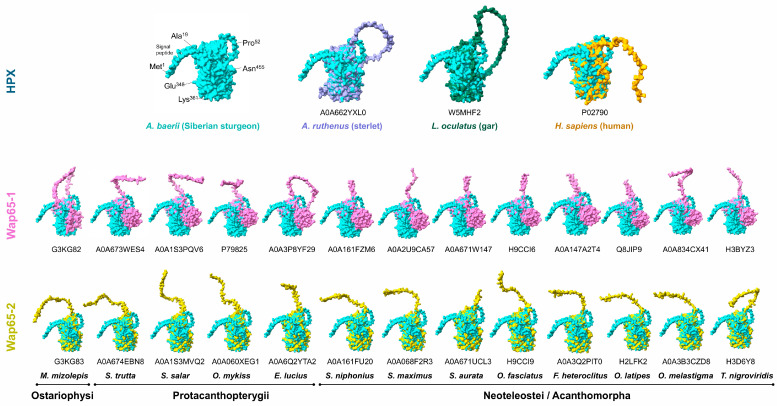
Structural superimposition of *A. baerii* HPX with representative vertebrate HPX and Wap65 isoforms. Unlike superimposition with Wap65-2, Wap65-1 displays a unique structurally divergent region in the C-domain across all comparisons, consistent with isoform-specific divergence following gene duplication. Protein IDs used for modeling and superimposition are shown for each sequence.

The predicted 3D structure of *Acipenser baerii* HPX was first compared with HPX orthologs from *Acipenser ruthenus* (sterlet) and *Lepisosteus oculatus* (gar). The model showed a high degree of structural similarity with *A. ruthenus* HPX, consistent with their close evolutionary relationship. The holostean HPX from *L. oculatus* also exhibited strong structural conservation with *A. baerii*, particularly in the arrangement of the two β-propeller-like domains (Figure 4). These findings support the phylogenetic positioning of sturgeon HPX as a basal member of the actinopterygian clade.

Further comparisons were made with representative teleost Wap65 isoforms. Superimposition of *A. baerii* HPX with multiple Wap65-2 models from species across Ostariophysi, Protacanthopterygii, and Neoteleostei showed consistent overall structural similarity. In contrast, all comparisons with Wap65-1 isoforms revealed a distinct structural deviation in the C-domain, where a non-aligned or uniquely diverged region was consistently observed. This suggests that the C-domain has undergone isoform-specific structural divergence in Wap65-1, differentiating it from the ancestral HPX fold retained in *A. baerii* (Figure 4).

### 2.5. Tissue Distribution of HPX mRNA Expression in Fingerling A. baerii

**Figure 5:**
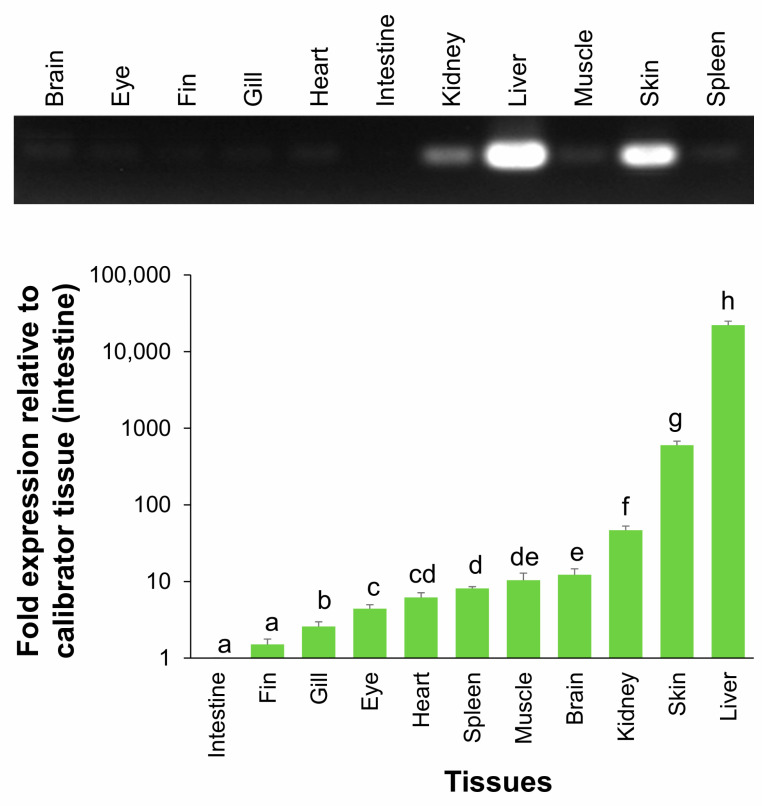
Tissue distribution patterns and expression levels of *HPX* mRNA in *A. baerii* fingerlings. Quantitative RT-PCR analysis of *HPX* transcript levels across 11 tissues from healthy fingerlings. Expression levels were normalized using the geometric mean of three reference genes (*RPL5*, *RPL7*, and *RPL7A*) and are presented as fold differences relative to the calibrator tissue (intestine). Data are shown as mean ± SD (*n* = 6; biological replications). Statistically significant differences among tissues were determined using one-way ANOVA followed by Tukey’s post hoc test. Different lowercase letters indicate significant differences (*p* < 0.05). A representative image of ethidium bromide-stained agarose gel (1%) from end-point RT-PCR amplification is also shown on the top. Expression data normalized with each reference control can be referred to Supplementary Figure S5.

To evaluate the tissue-specific expression profile of *HPX* in fingerling *Acipenser baerii*, RT-qPCR analysis was conducted using RNA extracted from multiple tissues. *HPX* mRNA was detected in all examined tissues, but transcript abundance varied markedly. The liver showed overwhelmingly dominant expression, followed by skin and kidney, whereas tissues such as intestine, fin, and gill exhibited only trace levels (Figure 5). Normalization using each of the three selected reference genes yielded consistent expression ranking among tissues, although the absolute fold differences varied slightly depending on the reference (Supplementary Figure S5). When the intestine—identified as the tissue with the lowest expression—was used as the calibrator, *HPX* expression in the liver averaged 22,128 ± 2690-fold higher. The expression levels in skin and kidney were also elevated, measured at 600.6 ± 77.2 and 46.8 ± 6.2-fold, respectively, compared to the intestine.

### 2.6. Ontogenetic Expression of HPX During Early Development in A. baerii

**Figure 6:**
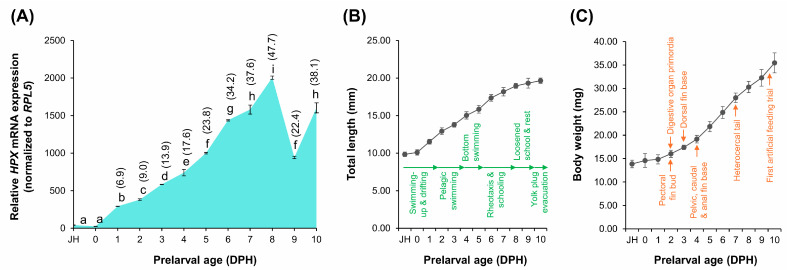
Ontogenetic expression pattern of *HPX* mRNA during prelarval intervals in *A. baerii*. (**A**) Relative *HPX* mRNA expression levels measured by RT-qPCR from the just-hatched (JH) stage through 10 days post-hatching (DPH), normalized to *RPL5*. Figures in parentheses are fold change of expression levels relative to that at JH. Different lowercase letters denote statistically significant differences among time points (*p* < 0.05, ANOVA with Tukey’s post hoc test), based on triplicate biological replications (each comprising of 3–4 pooled prelarvae). (**B**,**C**) Corresponding growth curves for total length (**B**) and body weight (**C**) over the same developmental window. Developmental milestones for behavior and metamorphosis are also indicated.

To examine the developmental regulation of *HPX* transcription, *HPX* mRNA levels were measured in *A. baerii* prelarvae and larvae from the just-hatched (JH) stage through 10 days post-hatching (DPH). During this period, the fish exhibited characteristic early life stage transitions, including fin and digestive primordia formation and behavioral shifts such as the pelagic-to-benthic transition and the onset of schooling behavior. Normalization was performed using *RPL5*, previously validated as a stable internal control for developmental samples. *HPX* expression remained minimal through the JH and 0 DPH stages but began to increase significantly from 1 DPH onward (*p* < 0.05). Expression continued to rise and peaked at 8 DPH. A marked, transient reduction was observed at 9 DPH, followed by partial recovery at 10 DPH (Figure 6A). This decline in *HPX* expression occurred despite continuous increases in total length and body weight throughout the same period (Figure 6B,C), indicating that the transcriptional fluctuation was not associated with general somatic growth.

### 2.7. Dose-Dependent Modulation of HPX Expression Following A. hydrophila Challenge in A. baerii Fingerlings

During the survival/mortality test across a range of *Aeromonas hydrophila* concentrations (2 × 10^4^ to 2 × 10^9^ CFU/g body weight), mortality occurred rapidly and was dose-dependent, with all deaths occurring within 36 h post-injection. Complete mortality was observed at 2 × 10^9^ CFU/g, while no significant mortality occurred at 2 × 10^4^ CFU/g and below. The 2 × 10^5^ CFU/g dose resulted in approximately 50% survival, indicating an LD_50_ near this level and defining 2 × 10^4^ CFU/g as the upper threshold of the sublethal range (Supplementary Figure S6).

**Figure 7:**
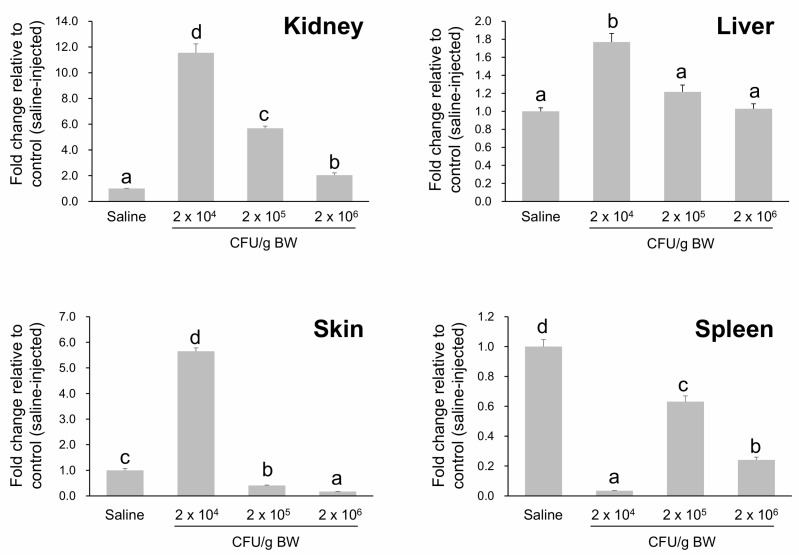
Differential expression of *HPX* mRNA in responsive to experimental infection with different doses of *A. hydrophila* in *A. baerii* fingerlings. Quantitative RT-PCR analysis of *HPX* transcript levels at 12 h post-injection (HPI) in kidney, liver, skin, and spleen tissues following intraperitoneal injection of *A. hydrophila* at 2 × 10^4^, 2 × 10^5^, or 2 × 10^6^ CFU/g body weight (BW). Expression values were normalized to *RPL5* and are shown as fold change relative to saline-injected controls. Data represent mean ± SD (*n* = 6). Statistically significant differences among dose groups within each tissue were evaluated using one-way ANOVA followed by Tukey’s test. Different letters indicate significant differences (*p* < 0.05).

To examine early transcriptional responses, *HPX* mRNA levels were measured 12 h post-injection (HPI) following exposure to three bacterial doses: 2 × 10^4^, 2 × 10^5^, and 2 × 10^6^ CFU/g. A clear dose-dependent pattern of *HPX* modulation was observed across tissues (Figure 7). In the kidney, *HPX* expression was strongly induced at 2 × 10^4^ CFU/g (12.6-fold relative to control) but decreased progressively at higher doses. The skin showed a similar trend, with peak induction at the lowest dose and marked downregulation at the highest dose. In the liver, a modest increase was observed at 2 × 10^4^ CFU/g, with no further upregulation at higher concentrations. Notably, *HPX* expression in the spleen was significantly suppressed at all tested doses, suggesting an immunosuppressive or exhaustion-related response. These results indicate that excessive bacterial burden can override the capacity for *HPX* upregulation, particularly in mucosal and hematopoietic tissues. Based on both survival data and gene expression profiles, the 2 × 10^4^ CFU/g dose was selected as an optimal sublethal challenge condition for subsequent immune response analyses.

### 2.8. Temporal Expression Dynamics of HPX and TF Following A. hydrophila Challenge

**Figure 8:**
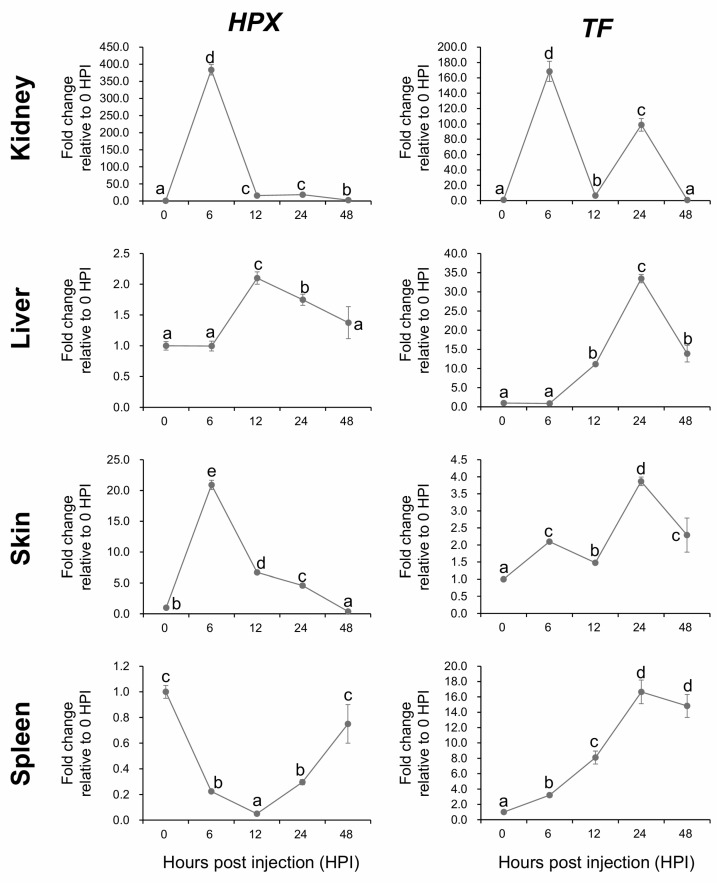
Temporal modulation of *HPX* and *TF* mRNA expression following *A. hydrophila* injection in *A. baerii* fingerling tissues (kidney, liver, skin and spleen), assessed at 0, 6, 12, 24, 48 h post-injection (HPI) based on RT-qPCR. Expression values were normalized to *RPL5* and are shown as fold change relative to 0 HPI. Data represent mean ± SD (*n* = 4). Different lowercase letters indicate significant differences among time points within each tissue (*p* < 0.05, ANOVA with Tukey’s test).

Following the initial dose optimization, the temporal patterns of *HPX* expression were examined in fingerling *A. baerii* during the early immune response to *A. hydrophila*. Transcript levels were measured at 0, 6, 12, 24, and 48 h post-injection (HPI), alongside expression of the known immune transcription factor transferrin (*TF*), across four tissues: kidney, liver, skin, and spleen (Figure 8).

In the kidney, *HPX* was rapidly and robustly upregulated, reaching a peak of nearly 400-fold relative to baseline at 6 HPI, before gradually decreasing at later timepoints, although remaining elevated above baseline throughout the period. This early upregulation closely mirrored the pattern observed for *TF*, which also peaked at 6 HPI with over 160-fold induction, followed by a second peak at 24 HPI. In contrast, *HPX* expression in the liver showed a delayed onset, with no significant upregulation at 6 HPI but reaching a peak at 12 HPI. Thereafter, expression steadily declined, returning to baseline by 48 HPI. The TF response in the liver was similarly delayed, with no early upregulation at 6 HPI and maximal induction observed at 24 HPI.

In the skin, *HPX* expression followed a pattern similar to that in the kidney, with strong induction at 6 HPI followed by a decline over time. *TF* in the skin was modestly induced at 6 HPI and showed a second, stronger peak at 24 HPI, again resembling the kidney pattern. In the spleen, the expression dynamics differed notably. *HPX* was rapidly downregulated following challenge, reaching its lowest level at 12 HPI and returning to baseline by 48 HPI. In contrast, TF expression in the spleen steadily increased, peaking at 24 HPI. This divergence suggests that spleen-specific regulation of *HPX* may involve immune suppression or exhaustion mechanisms distinct from those influencing *TF* (Figure 8).

### 2.9. HPX Expression Under Mild and Severe Thermal Stress in A. baerii Fingerlings

**Figure 9:**
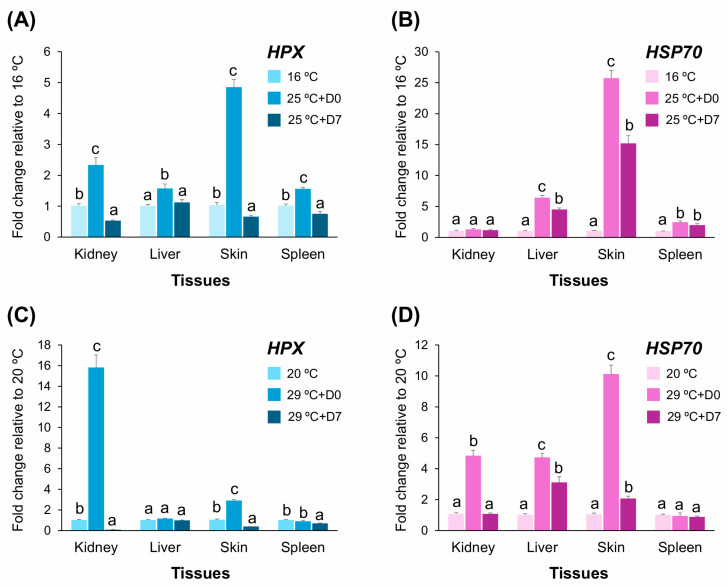
Tissue-specific expression of *HPX* and *HSP70* under mild and severe thermal stress in *A. baerii* fingerlings. (**A**,**B**) Fold change in *HPX* (**A**) and *HSP70* (**B**) mRNA expression in kidney, liver, skin, and spleen after exposure to mild thermal stress (16 °C to 25 °C). Expression was measured immediately after reaching the target temperature elevation (25 °C + D0) and after additional exposure at 25 °C for 1 week (25 °C + D7). (**C**,**D**) *HPX* (**C**) and *HSP70* (**D**) mRNA expression following severe thermal stress (20 °C to 29 °C) at the same time points (20 °C, 29 °C + D0 and 29 °C + D7). Gene expression levels were normalized to RPL5 and shown as fold change relative to baseline temperatures (16 °C or 20 °C). Data are shown as mean ± SD (*n* = 4). Different lowercase letters denote statistically significant differences within each tissue across treatments (*p* < 0.05, ANOVA with Tukey’s post hoc test).

To compare transcriptional responses of *HPX* under high-stress temperature conditions, fingerling *A. baerii* were exposed to two thermal regimes: (1) a mild elevation from 16 °C to 25 °C, and (2) a severe elevation from 20 °C to 29 °C. Gene expression was assessed in the kidney, liver, skin, and spleen at baseline, immediately after temperature increase (D0), and after one week of continuous exposure (D7). *HPX* expression was compared alongside HSP70, a canonical thermal stress marker (Figure 9A–D).

Under the mild treatment (16 °C → 25 °C), *HPX* expression was upregulated in all four tissues at D0. The skin showed the highest induction (4.85-fold), followed by moderate increases in the kidney (2.3-fold), liver, and spleen (each <2-fold). However, by D7, *HPX* expression decreased markedly in all tissues—dropping below baseline in kidney and skin and returning to near-control levels in liver and spleen. In contrast, *HSP70* expression showed more sustained activation. While the skin exhibited a dramatic increase (>25-fold at D0 and >15-fold at D7), liver and spleen also maintained significantly elevated expression at D7. The kidney showed no significant *HSP70* response, highlighting a divergence from *HPX* activation patterns (Figure 9A,B).

Under the severe treatment (20 °C → 29 °C), *HPX* was highly induced in the kidney at D0 (15.8-fold) but sharply downregulated at D7 (<0.1-fold). In the skin, a 2.9-fold increase at D0 was followed by near-complete suppression at D7. In liver and spleen, *HPX* expression remained largely unresponsive or suppressed throughout. *HSP70* expression, in contrast, was broadly upregulated across kidney, liver, and skin at D0, and remained elevated (particularly in liver) through D7. The spleen showed only modest *HSP70* activation (Figure 9C,D).

## 3. Discussion

### 3.1. Structural Features and Evolutionary Context of A. baerii HPX

The structural analysis of *A. baerii* HPX confirms that it retains all canonical features typical of vertebrate hemopexins. Conserved heme-binding histidines, multiple metal-binding residues, disulfide-forming cysteine pairs, and a predicted β-propeller-like domain organization strongly suggest that this ancestral form remains structurally competent. These motifs are critical for heme sequestration and transport, supporting the preservation of detoxification potential in the sturgeon lineage.

While it is well established that teleost fish possess two HPX-derived paralogs, Wap65-1 and Wap65-2, sequence alignment and similarity analyses in this study clearly position *A. baerii* HPX closer to Wap65-2. This pattern is consistent with previous studies identifying Wap65-2 as the isoform more functionally aligned with mammalian HPX, particularly in heme-binding and immune-responsive roles [8,9,27]. This relationship is further supported by the conservation of key residues, including the two canonical heme-binding histidines. In human HPX, at least four histidines have been implicated in heme coordination; however, only two are consistently recognized as essential for direct and high-affinity binding, while others are likely to play flexible or condition-dependent roles [28,29]. The retention of these two critical residues in *A. baerii* HPX—at positions directly corresponding to their human counterparts—underscores the conserved functional core preserved across distant vertebrate lineages.

The structural comparison further emphasizes the divergence between ancestral HPX and teleost Wap65-1. Three-dimensional superimposition revealed high congruence between *A. baerii* HPX and other vertebrate HPX/Wap65-2 orthologs, particularly in the spatial arrangement of binding residues and overall domain organization. In contrast, Wap65-1 consistently exhibited a structurally divergent region in the C-domain that failed to align with the HPX scaffold. This C-domain modification may reflect isoform-specific structural remodeling associated with post-duplication neofunctionalization and/or thermal specialization in teleost lineages [10].

Phylogenetic analyses reinforce this interpretation. Both maximum likelihood (ML) and neighbor-joining (NJ) trees consistently placed *A. baerii* HPX basal to the teleost Wap65-1 and Wap65-2 clades, forming a distinct lineage alongside the holostean *L. oculatus*. Although *A. baerii* HPX shares significantly greater sequence identity and similarity with teleost Wap65-2 than with Wap65-1, it did not cluster within either paralogous clade. This reflects the fact that phylogenetic topology captures deeper evolutionary divergence patterns, beyond simple pairwise similarity. Importantly, the sturgeon HPX was resolved within the basal actinopterygian radiation, clearly separated from sarcopterygian HPXs and the neopterygian Wap65 paralogs. Based on current phylogenomic estimates, Chondrostei (e.g., sturgeons) diverged from Neopterygii approximately 360–330 million years ago, while Holostei (e.g., gars) split from Teleostei later, around 300–250 million years ago. The teleost-specific third round of whole-genome duplication (3R WGD), which gave rise to Wap65-1 and Wap65-2, likely occurred after these divergences, approximately around 210–200 million years ago [30,31,32].

Together, these lines of evidence support the conclusion that *A. baerii* HPX represents a pre-duplication, ancestral form of vertebrate hemopexin. Furthermore, sturgeons are known to exhibit unusually slow molecular evolution, with significantly lower substitution rates than most teleosts across nuclear loci [11,33]. This slow evolving genomic context, combined with the early phylogenetic position and retention of a single-copy *HPX* gene, suggests that *A. baerii HPX* preserves an evolutionarily informative prototype for understanding ancestral structural and functional features from which the teleost Wap65 paralogs diverged through sub- and neofunctionalization

### 3.2. Basal Expression Patterns of HPX in Tissues and Early Ontogenic Development

The basal expression profile of *A. baerii HPX* shows distinct tissue-specific patterns, with particularly high transcript abundance in the liver, followed by the skin and kidney. Its predominant expression in the liver aligns with both mammalian *HPX* and teleostean *Wap65*, reinforcing a conserved function in systemic heme detoxification and redox regulation. However, considerable *HPX* mRNA levels in the skin and kidney—mucosal and excretory tissues exposed to external stressors—suggest its additional roles in localized functions associated with oxidative defense or barrier protection [34,35]. Meanwhile, among diverged teleostean lineages, tissue expression patterns of Wap65 isoforms have shown considerable variation, indicating species- or lineage-specific regulation rather than a conserved expression framework—barring the consistent and predominant hepatic expression observed across both *Wap65* isoforms [8,9,36,37].

During early development, *A. baerii HPX* expression increased progressively from hatching to 8 DPH. This ontogenic rise coincides with key behavioral and physiological transitions, including the development of fins, digestive organs, and elevated swimming activity. From 1 to 3 DPH, prelarvae exhibit phototactic pelagic swimming and heightened locomotor activity, likely associated with increased oxygen consumption and reactive oxygen species (ROS) production [24]. Between 5 and 8 DPH, larvae transition to benthic swimming, characterized by strong rheotaxis and active schooling—also energetically demanding behaviors [24,38]. The sustained upregulation of *HPX* may reflect anticipatory protection against oxidative stress linked to these high-energy phases and to the expansion of vascular structures, including branchial circulation and external gill development [39,40].

These patterns support the interpretation that *HPX* functions as a developmentally regulated oxidative buffer—not limited to hemolysis or infection but integrated into the broader physiological adaptations required during early life-stage transitions. Notably, the ontogenetic increase of *A. baerii HPX* expression mirrors the early developmental upregulation of *Wap65-2* reported in several teleosts [8,41,42], suggesting a functional persistence of redox-regulatory or protective roles. In contrast, *Wap65-1* tends to show less consistent activation during early development, reinforcing the closer expression alignment of *HPX* with *Wap65-2*.

A transient decline in *HPX* expression was observed at 9 DPH, despite continued increases in total length and body weight. This stage coincides with the developmental transition from exclusive reliance on endogenous yolk reserves to the initiation of exogenous feeding. At this point, prelarvae evacuate the yolk plug and begin ingesting artificial diets, a process that involves substantial metabolic and physiological reorganization. Behaviorally, this period is often marked by reduced locomotion (“post-schooling rest”) [38], and similar suppression has been reported for multiple immune-related genes at this interval [14,39]. These findings suggest that *HPX* expression during development is modulated in a non-linear fashion, likely reflecting shifts in metabolic and physiological priorities—particularly the transition from endogenous and exogenous nutrient utilization in early development.

Taken together, the liver-dominant yet extrahepatic tissue expression, combined with ontogenetic regulation during key transitional phases, supports the view that *A. baerii HPX* contributes to baseline physiological homeostasis in multiple tissues. These roles likely include systemic heme clearance, localized oxidative stress buffering, and early-life adaptation to rising metabolic demands.

### 3.3. Multivalent Stress Responsiveness: HPX Under Infection and Heat Challenge

The challenge experiments demonstrate that *A. baerii HPX* expression is dynamically modulated by both immune and thermal stresses. Under *A. hydrophila* challenge, *HPX* showed a dose- and tissue-dependent regulation [25,26]. At sublethal doses, strong upregulation occurred in the kidney and skin—tissues involved in immune surveillance and hemolysis mitigation—while the liver and spleen exhibited only modest or even suppressed responses. At higher bacterial loads, *HPX* expression decreased or became inhibited, particularly in the spleen, suggesting an exhaustion-like response under overwhelming stress [14,43,44].

The temporal regulation of *HPX* revealed further tissue-specific dynamics. Kidney and skin showed rapid and strong induction at 6 h post-injection, consistent with early-phase, local immune activation at barrier sites. Liver upregulation was delayed, reflecting relatively slower, systemic acute-phase signaling [45,46]. In contrast, splenic expression of *HPX* was transiently downregulated despite continued upregulation of *TF*, indicating gene-specific regulatory divergence during immune activation. Although the precise basis for this pattern remains unclear, it may involve tissue-specific transcriptional controls, differences in inflammatory sensitivity, or compartmentalized immune signaling [47,48]. Overall, *HPX* appears to function as a context-sensitive acute-phase protein, with both systemic and peripheral roles varying by tissue and stimulus intensity.

In thermal stress experiments, both mild (16 °C → 25 °C) and severe (20 °C → 29 °C) temperature shifts induced *HPX* expression at the acute phase (D0). The strongest response occurred in the skin (mild stress) and kidney (severe stress). However, in the liver—where teleost *Wap65-1* is typically upregulated under thermal stress—*HPX* levels remained unchanged or minimally altered, unlike the robust hepatic induction of *HSP70*. This suggests that the thermal responsiveness of *HPX* in *A. baerii* is localized and non-systemic, likely linked to transient oxidative imbalance or inflammation in peripheral tissues rather than coordinated hepatic regulation [49,50].

However, the elevated expression at D0 markedly declined or even suppressed at D7. Compared to relatively sustained expression of *HSP70*, the downregulation of *HPX* occurred more rapidly during thermal acclimation, supporting its role as an early-phase protective or compensatory factor. Previous studies have noted that chronic thermal stress disrupts immune regulation and physiological balance in Acipenseriform species, with species- and context-specific variation in stress responses [17,51,52]. While *HPX* plays a role during the acute heat response, its transient expression suggests a transition to other long-term stress management systems during thermal adaptation.

While our qPCR-based analysis provides robust and reproducible quantification of *HPX* transcripts, it is limited to mRNA-level measurements and does not address potential post-transcriptional or post-translational regulation. In the polyploid genomic context of *A. baerii*, the possibility of undetected paralogous loci or sequence variants cannot be fully excluded, although the inclusion of conserved-region primer testing supports the reliability of our quantification. Future work incorporating protein-level assays (e.g., Western blotting, targeted proteomics) and functional studies will be important to further elucidate the regulatory mechanisms and physiological roles of *HPX* in sturgeons.

Taken together, *HPX* exhibits transcriptional responsiveness to both bacterial and thermal stimuli, with stressor- and tissue-specific modulation. While its immune-related regulation is robust and evident across multiple tissues, thermal sensitivity is more modest and localized. These findings support the view that ancestral *HPX* possessed multivalent stress reactivity, with immune-regulatory function as the dominant feature and heat responsiveness as a supplementary, context-dependent trait. This functional plasticity is consistent with the hypothesis that ancestral *HPX* functions were partitioned following *Wap65* gene duplication in teleosts, with *Wap65-1* evolving toward a specialized, heat-responsive role.

## 4. Materials and Methods

### 4.1. Molecular Cloning of Acipenser baerii Hemopexin (HPX) cDNA

Putative *HPX/Wap65*-related sequences were identified from a local *A. baerii* transcriptomic resource comprising paired-end Illumina short-read assemblies and full-length transcript isoforms generated via PacBio Iso-Seq from multiple tissue sources. Contigs showing high similarity to known vertebrate hemopexin (*HPX*) or warm-temperature acclimation-associated 65 kDa protein (*Wap65*) orthologs were assembled using Unipro UGENE software v52.1 (Unipro, LLC, Novosibirsk, Russia; https://ugene.net/, accessed on 13 August 2025).To isolate the presumed full-length open reading frame (ORF), gene-specific primers targeting the 5′- and 3′-untranslated regions (UTRs) were designed based on assembled contigs. PCR amplification was performed using 0.5 μg of total RNA extracted from the liver of a female *A. baerii* as the template. Oligonucleotide primer sequences are listed in Supplementary Table S1. The amplified product was visualized by electrophoresis on a 1% agarose gel stained with ethidium bromide. A single specific band was gel-purified and sequenced bidirectionally to obtain the representative cDNA sequence of *A. baerii HPX*.

### 4.2. Bioinformatic Sequence Analysis

#### 4.2.1. Sequence Characterization

The open reading frame (ORF) of *A. baerii HPX* was identified using the ORF Finder tool (National Center for Biotechnology Information, Bethesda, MD, USA; https://www.ncbi.nlm.nih.gov/orffinder/, accessed on 13 August 2025), and the deduced amino acid sequence was analyzed with the ExPASy ProtParam tool (Swiss Institute of Bioinformatics, Lausanne, Switzerland; https://web.expasy.org/protparam/, accessed on 13 August 2025) to calculate molecular weight and theoretical isoelectric point (pI). The signal peptide and its predicted cleavage site were assessed using SignalP 6.0 (v6.0; Technical University of Denmark, Lyngby, Denmark; https://services.healthtech.dtu.dk/services/SignalP-6.0/, accessed on 13 August 2025). Conserved domain architecture and structural features—including heme-binding, metal-binding, and disulfide-forming motifs—were annotated using InterPro (European Bioinformatics Institute, Cambridge, UK; https://www.ebi.ac.uk/interpro/, accessed on 13 August 2025), SMART (European Molecular Biology Laboratory, Heidelberg, Germany; http://smart.embl-heidelberg.de/, accessed on 13 August 2025), and the NCBI Conserved Domain Database (CDD; National Center for Biotechnology Information, Bethesda, MD, USA; https://www.ncbi.nlm.nih.gov/Structure/cdd/wrpsb.cgi, accessed on 13 August 2025). Potential N-glycosylation sites were predicted using NetNGlyc 1.0 (v1.0; Technical University of Denmark, Lyngby, Denmark; https://services.healthtech.dtu.dk/services/NetNGlyc-1.0/, accessed on 13 August 2025).

#### 4.2.2. Multiple Sequence Alignments and Molecular Phylogeny

A total of 79 HPX/Wap65 protein sequences from 46 vertebrate species were retrieved from GenBank based on BLASTP homology search (National Center for Biotechnology Information, Bethesda, MD, USA; https://blast.ncbi.nlm.nih.gov/Blast.cgi, accessed on 13 August 2025). These included three cartilaginous fishes (Chondrichthyes), ten sarcopterygian tetrapods, one holostean, two chondrosteans (including *A. baerii*), and 31 teleosts, each represented by two Wap65 isoforms where available (Supplementary Table S2). Due to inconsistent nomenclature in public databases, sequence inclusion was based on alignment similarity rather than annotated gene names. Multiple sequence alignments were performed using ClustalW (Kyoto University Bioinformatics Center, Kyoto, Japan; https://www.genome.jp/tools-bin/clustalw, accessed on 13 August 2025) to assess conserved regions and diagnostic motifs. Pairwise identity and similarity were calculated using the Ident and Sim tool from the Sequence Manipulation Suite v2 (University of Alberta, Edmonton, AB, Canada; https://www.bioinformatics.org/sms2/, accessed on 13 August 2025).

Phylogenetic trees were constructed using MEGA v12 (Temple University, Philadelphia, PA, USA; Tokyo Metropolitan University, Tokyo, Japan; https://www.megasoftware.net/). Maximum likelihood (ML) analysis was conducted under the JTT model with gamma distribution and invariant sites (G+I), using all alignment sites. Heuristic search was performed with the nearest-neighbor interchange (NNI) method. Node confidence was evaluated via both adaptive bootstrap (threshold = 5.0) and conventional bootstrap (1000 replicates). For comparison, neighbor-joining (NJ) trees were reconstructed under different substitution models (JTT, Poisson, Dayhoff, and p-distance), rate heterogeneity settings (uniform and gamma-distributed), and gap/missing data strategies (pairwise and complete deletion). NJ topologies were supported by 1000 bootstrap replicates.

#### 4.2.3. Three-Dimensional Structural Modeling and Superimposed Analysis

The full-length amino acid sequence, including the signal peptide, was submitted to ColabFold v1.55 (an AlphaFold2-based platform using MMseqs2; Max Planck Institute for Multidisciplinary Sciences, Göttingen, Germany; https://colab.research.google.com/github/sokrypton/ColabFold/blob/main/AlphaFold2.ipynb, accessed on 13 August 2025) to generate de novo 3D structural models. Structural confidence was evaluated by pLDDT scores, MSA coverage, and predicted aligned error (PAE) plots for the top five ranked models. The highest-ranking structure (PDB format) was visualized and annotated using UCSF ChimeraX v1.10 (University of California, San Francisco, CA, USA; https://www.rbvi.ucsf.edu/chimerax), enabling exploration of domain features, loops, and functional motifs.

Structural superimposition was performed using the MatchMaker tool in ChimeraX, comparing *A. baerii* HPX with homologous HPX and Wap65 structures from other vertebrates, obtained from the AlphaFold Protein Structure Database (European Bioinformatics Institute, Cambridge, UK; https://alphafold.ebi.ac.uk/, accessed on 13 August 2025). Conserved features including heme-binding histidines, cysteine pairs, metal-binding motifs, and glycosylation sites were examined for spatial conservation and divergence.

### 4.3. Tissue and Prelarval Sampling for Basal Expression Analysis

To assess the tissue-specific distribution of *HPX* mRNA, eleven somatic tissues (brain, eye, fin, gill, intestine, heart, kidney, liver, muscle, skin, and spleen) were collected from healthy *A. baerii* fingerlings (*n* = 6; average total length = 10.5 ± 1.8 cm). Fish were euthanized with buffered MS-222 (tricaine methanesulfonate; 1000 ppm; Sigma-Aldrich, St. Louis, MO, USA) and sampled. All individuals were artificially bred by hormone-induced spawning and reared under standard aquaculture conditions (19–20 °C) in a semi-recirculating freshwater system at the Experimental Fish Culture Station, Pukyong National University (PKNU), Busan, South Korea. Immediately upon excision, tissue samples were snap-frozen in dry ice and stored at −85 °C until RNA extraction.

For ontogenetic expression analysis, prelarvae were reared under the same environmental conditions from hatching through 10 days post-hatching (DPH), encompassing the endogenous-to-exogenous feeding transition. Morphometric traits (wet body weight to the nearest 0.1 mg and total length to the nearest 0.01 mm) were measured daily, and key developmental milestones (e.g., fin formation, phototaxis, rheotaxis) were recorded. At each time point, three biological replicates were collected, each comprising pooled whole-body samples of 3–4 randomly selected prelarvae. Samples were immediately frozen in dry ice and stored at −85 °C for subsequent RNA-based analysis.

### 4.4. Bacterial and Thermal Challenges for Differential Expression Analyses

#### 4.4.1. Bacterial Challenge

Healthy *A. baerii* fingerlings (average total length = 11.4 ± 2.1 cm), reared at the PKNU Experimental Fish Culture Station under standard conditions (see Section 4.3), were used for all bacterial challenge experiments. Prior to the trial, absence of *Aeromonas* species was confirmed in the tank water and fish tissue samples (*n* = 6) using *Aeromonas*-specific isolation agar (Millipore; Merck KGaA, Darmstadt, Germany). All experimental tanks received filtered (1.0 μm) groundwater (pH 7.0–7.3; NH_3_ < 0.05 mg/L) at 19 ± 0.5 °C, with dissolved oxygen maintained between 6 and 7 mg/L using both air blowers and supplemental oxygenation.

The challenge pathogen, *Aeromonas hydrophila* (KCTC 2358; Gram-negative), was administered via intraperitoneal (IP) injection in saline (100 μL) at doses ranging from 2 × 10^4^ to 2 × 10^9^ CFU/g body weight (10-fold intervals). Each dose group (*n* = 12) was housed in a 100 L aerated tank (0.5 × 1.0 × 0.3 m) with independent sand filtration. A saline-injected control group was identically prepared. Post-injection mortality was monitored at 2 h intervals for 48 h.

To assess dose-dependent *HPX* expression, three bacterial concentrations were selected based on the survival test: a sublethal dose (2 × 10^4^ CFU/g), approximate LD_50_ (2 × 10^5^ CFU/g), and a high-lethality dose (2 × 10^6^ CFU/g). At 12 h post-injection (HPI), liver, kidney, skin, and spleen tissues were collected from six randomly selected individuals per group, immediately frozen, and stored at −85 °C for gene expression analysis. For temporal expression profiling, fish were injected at 2 × 10^4^ CFU/g (sublethal dose) as above. Tissue samples (liver, kidney, skin, spleen) were collected at 0, 6, 12, 24, and 48 HPI from four randomly selected individuals per time point. The 0 h group received saline injection and served as baseline control.

#### 4.4.2. Thermal Treatment

Two consecutive thermal challenge experiments were conducted using fingerlings previously acclimated to lower baseline temperatures. In the first experiment (mild stress), fish were pre-acclimated at 16 °C for 7 days and then subjected to a gradual thermal increase to 25 °C—considered non-lethal but physiologically stressful under prolonged exposure. Two replicate tanks (*n* = 12 fish per tank) were used for the thermal elevation group (16 °C → 25 °C; 1 °C increase per 3 h), while two control tanks (*n* = 12 each) were maintained at 16 °C. Tank size and environmental parameters were consistent with those used in bacterial challenge experiments, except for the temperature variable. For gene expression analysis, four individuals were randomly sampled from each tank at three time points: (1) prior to heat ramping (baseline at 16 °C), (2) immediately after reaching 25 °C (designated 25 °C + D0), and (3) following 7-day exposure at 25 °C (designated 25 °C + D7). Tissues collected included liver, kidney, skin, and spleen, and stored at −85 °C for gene expression analysis.

In the second experiment (severe stress), a separate group of fingerlings maintained at 20 °C was subjected to thermal elevation to 29 °C. This temperature is known to be highly stressful for *A. baerii* fingerlings, inducing physiological dysregulation and potential mortality upon prolonged exposure. Although no mortality occurred during the experiment, fish exhibited abnormal bottom-dwelling and reduced locomotor activity. Two replicate treatment tanks (29 °C) and two control tanks (20 °C) were prepared as described above. Sampling was conducted at baseline (at 20 °C), immediately after reaching 29 °C (29 °C + D0), and following 7-day exposure at 29 °C (29 °C + D7), using four randomly selected individuals per tank. Same kinds of tissues (liver, kidney, skin, and spleen) were collected.

### 4.5. Nucleic Acid Preparation and RT-qPCR

#### 4.5.1. RNA Extraction and cDNA Synthesis

Total RNA was extracted using TRIzol reagent (Thermo Fisher Scientific, Waltham, MA, USA), followed by on-column purification and DNase treatment with the RNeasy Mini Kit (Qiagen, Hilden, Germany) according to the manufacturers’ protocols. RNA concentration and quantity were determined spectrophotometrically at 260 nm using a NanoDrop ND-1000 (Thermo Fisher Scientific). RNA purity was assessed based on A260/A280 and A260/A230 ratios, with acceptable values ≥1.9 and ≥2.0, respectively.

For reverse transcription, 2 μg of total RNA was used to synthesize cDNA with the Omniscript RT Kit (Qiagen), using a primer mix consisting of oligo(dT)_20_ and random nonamers according to manufacturer’s instruction. Resulting cDNA products were diluted 10-fold in nuclease-free water and stored at −85 °C until used as templates for quantitative PCR.

#### 4.5.2. Reference Gene Selection and Quantitative PCR Conditions

To select stable internal reference genes for normalization, eight candidate genes were tested across multiple tissue types: β-2-microglobulin (*B2M*), ribosomal protein L4 (*RPL4*), ribosomal protein L7A (*RPL7A*), succinate dehydrogenase complex flavoprotein subunit A (*SDHA*), ubiquitin-conjugating enzyme (*UBE*), glyceraldehyde-3-phosphate dehydrogenase (*GAPDH*), ribosomal protein L5 (*RPL5*), and ribosomal protein L7 (*RPL7*). RPL5, RPL7, and RPL7A were identified as the most stable, each showing a coefficient of variation (%CV) below 3% in CT values (unpublished data). To ensure robust and reproducible quantification of *HPX* transcripts, three primer pairs were evaluated: one study-specific pair designed from the *A. baerii HPX* sequence obtained in this study for use across all experimental samples, and two pairs designed at conserved regions of publicly available *Acipenser HPX*-like sequences to maximize inclusiveness across potential interspecific or allelic variants. The study-specific pair was applied to all samples, whereas the two conserved pairs were tested on a representative subset of 15 cDNA samples spanning four experimental sets to validate cross-lineage amplification performance. Reliability was assessed by comparing relative quantification (RQ) values between primer pairs for the same samples, yielding high concordance correlation coefficients (CCC > 0.99) and strong linear relationships (Pearson’s *r* ≥ 0.99) with regression slopes close to 1.0. These results indicate that all tested primer pairs produce highly comparable quantification across diverse tissue types and experimental conditions. Full methodological and result details of the validation experiments are provided in Supplementary Figure S7 and Table S3.

Quantitative PCR reactions were performed in a LightCycler 480 Real-Time PCR System (Roche Applied Science, Penzberg, Germany) using the LightCycler 480 SYBR Green I Master Mix. Each reaction contained 2 μL of diluted cDNA and 5 μM (final concentration) of each forward and reverse primer. Primer efficiency for all gene targets, including *HPX* and normalization controls, was confirmed to range from 92 to 102%, based on standard curves generated from five log-fold serial dilutions of pooled cDNA.

All qPCR assays were conducted in technical triplicates. Mean CT values were calculated per sample and used to compute biological replicates. Relative transcript levels for tissue distribution and ontogenetic expression were calculated using the 2^−ΔCT^ method [48,49]. For bacterial and thermal challenge experiments, fold changes in expression relative to control groups were determined using the 2^−ΔΔCT^ method [53,54].

### 4.6. Statistical Analysis

Statistical analyses were conducted using IBM SPSS Statistics software (version 29.0.2; IBM Corp., Armonk, NY, USA). Differences among group means for amino acid sequence identity/similarity, prelarval growth parameters (total length and body weight), and gene expression levels were assessed by one-way analysis of variance (ANOVA), followed by Tukey’s honestly significant difference (HSD) post hoc test. Statistical significance was accepted at *p* < 0.05. Survival differences among groups exposed to various bacterial challenge doses were evaluated using the Kaplan–Meier survival analysis with log-rank test, as implemented in the same software package. Statistical significance for survival comparisons was also set at *p* < 0.05.

## 5. Conclusions

This study provides the integrative characterization of hemopexin (*HPX*) in the Siberian sturgeon (*Acipenser baerii*), a basal actinopterygian lineage. The encoded protein retains conserved structural features, including motifs for heme binding, metal coordination, and disulfide bonding, and shows high structural similarity to mammalian HPX and teleost Wap65-2, but not to Wap65-1. Phylogenetic and 3D structural analyses position sturgeon HPX within a distinct basal clade of the Actinopterygian lineage, representing a pre-duplication prototype of the HPX/Wap65 family. Expression profiling revealed dominant hepatic transcription, strong immune-responsive induction, and modest, tissue-specific thermal sensitivity. These patterns indicate a multivalent but predominantly immune-related functional role, with thermal responsiveness more likely representing a component of a broader immune–stress response than a primary specialization. While our results are compatible with the possibility that *Wap65*-1 diverged from an ancestral *HPX* through sub- and/or neofunctionalization, potentially incorporating limited thermal responsiveness into its functional repertoire, this interpretation remains speculative and requires further evidence to confirm. The immune-related activities observed here are more directly supported by our data. Sturgeon *HPX* thus offers an evolutionarily informative reference for understanding the structural and regulatory foundations from which *HPX/Wap65* paralogs diversified in teleost fishes.

## Data Availability

The cDNA sequence of *Acipenser baerii* HPX generated in this study has been deposited in GenBank under accession number (PV871511).

## Supplementary Materials

The following supporting information can be downloaded at: https://www.mdpi.com/article/10.3390/ijms26167934/s1.

## Abbreviations

HPX	Hemopexin
Wap65	Warm-temperature acclimation-associated 65 kDa protein
TF	Transferrin
HSP70	Heat shock protein 70
RPL5	Ribosomal protein L5
RPL7	Ribosomal protein L7
RPL7A	Ribosomal protein L7A
DPH	Days post-hatching
HPI	Hours post-injection
CFU	Colony-forming unit
CT	Threshold cycle
