# Supplementary material for: Molecular Characterization of Hemopexin in the Siberian Sturgeon (Acipenser baerii): Evolutionary Insights and Differential Expression Under Immune and Thermal Stresses
Source: Int J Mol Sci. 2025 Aug 17;26(16):7934. doi: 10.3390/ijms26167934 (PMC12386703; doi:10.3390/ijms26167934)
Supplement: Supplementary file 1 [file ijms-26-07934-s001.zip › Suppl Table S3-Statistics of primer pair validation.pdf]

Supplementary Table S3. Statistical agreement between RQ values obtained with different *HPX* primer pairs. Primer pair #1 is the study-specific primers designed based on *Acipenser baerii* HPX sequences, while primer pairs #2 and #3 are designed at conserved-region across publicly available HPX sequences from *Acipenser* species. CI: 95% confidence interval; LOA: limits of agreement from Bland–Altman analysis; TOST: two one-sided tests for equivalence, bounds set at ±20% on the fold-change scale. Fold bias is the antilog_{10} of the mean bias value, with percentage difference shown in parentheses. Primer sequences are listed in **Table S1**

**Parameter**	**Primer pair #1 vs. pair #2**	**Primer pair #1 vs. pair #3**
**Pearson's correlation coefficient (*r*)**	0.99974	0.99896
**Lin's concordance correlation coefficient (CCC)**	0.99963	0.99891
**Deming regression**		
Slope (95% Confidence Interval; CI)	0.9970 (0.972 ~ 1.022)	1.0102 (0.998 ~ 1.022)
Intercept (95% Confidence Interval; CI)	−0.0077 (−0.097 ~ 0.081)	−0.0338 (−0.082 ~ 0.014)
**Bland–Altman analysis**		
Mean bias ($\log_{10}$ units)	−0.0168	−0.00239
Fold bias	0.962× (~3.8% lower)	0.995× (~0.5% lower)
95% Limit of Agreement (LOA; fold scale)	0.854× to 1.084×	0.779× to 1.270×
**TOST (two one-sided test) equivalence within ±20%**	Yes (p < 0.001)	Yes (p < 0.001)
