# Supplementary material for: Molecular Characterization of Hemopexin in the Siberian Sturgeon (Acipenser baerii): Evolutionary Insights and Differential Expression Under Immune and Thermal Stresses
Source: Int J Mol Sci. 2025 Aug 17;26(16):7934. doi: 10.3390/ijms26167934 (PMC12386703; doi:10.3390/ijms26167934)
Supplement: Supplementary file 1 [file ijms-26-07934-s001.zip › Suppl Fig Legends.pdf]

Supplementary Figure Legends

Supplementary Figure S1. Comparative analysis of sequence identity and similarity between *A. baerii* HPX and representative vertebrate HPX, Wap65-1, and Wap65-2 orthologs. Summarized percent amino acid identity and similarity between *A. baerii* HPX and orthologous sequences categorized by isoform and taxonomic group. Data were calculated using the Sequence Manipulation Suite (https://www.bioinformatics.org/sms2/ident_sim.html). Average identity and similarity values were compared across HPX, Wap65-1, and Wap65-2 clades. *A. baerii* HPX showed significantly higher similarity to Wap65-2 than to Wap65-1 ($p < 0.05$). Taxonomic breakdowns are shown for teleost subgroups (Ostariophysi, Protacanthopterygii, Stomiatii, Neoteleostei), non-teleost actinopterygians (e.g., Holostei), sarcopterygians (tetrapods), and cartilaginous fishes. Sequence data from other Acipenseriformes were excluded from statistical summaries to avoid bias from high intra-lineage similarity.

Supplementary Figure S2. Structural modeling quality assessment of *A. baerii* HPX using AlphaFold2 (ColabFold). **(A)** Sequence coverage and identity matrix used in the multiple sequence alignment (MSA) generation step, with individual sequences plotted by their identity to the query. **(B)** Predicted IDDT (Local Distance Difference Test) scores per residue across five ranked models, showing high-confidence predictions (>90) for most of the structure except the N-terminal signal region. **(C)** Predicted aligned error (PAE) heatmaps for five ranked models, with low inter-residue error in most regions, indicating high structural reliability.

Supplementary Figure S3. Structural comparison of conserved motifs in *A. baerii* HPX and human HPX.
(A) Superimposed structure highlighting conserved heme-binding histidine residues. *A. baerii* HPX contains two predicted key heme-binding histidines (His^{255} and His^{302}), corresponding to His^{236} and His^{293} in human HPX (P02790). **(B)** Superimposition of metal-binding motifs, showing eight conserved metal-binding sites distributed across N- (left) and C- (right) domains. Structural alignment reveals close spatial correspondence between sturgeon and human residues. **(C)** Conserved disulfide-forming cysteine residues. All five predicted cysteine pairs in *A. baerii* HPX structurally align with their human counterparts, supporting preservation of disulfide bridge architecture. **(D)** Comparison of predicted N-glycosylation sites. While several glycosylation sites are predicted in both species, only two (Asn^{206} and Asn^{455} in sturgeon, corresponding to Asn^{187} and Asn^{453} in human HPX) are conserved in location. Sturgeon-specific sites showed moderate potential (NetNGlyc scores 0.525–0.7288).

Supplementary Figure S4. Neighbor-joining (NJ) phylogenetic trees showing the placement of *A. baerii* HPX among vertebrate HPX/Wap65 orthologs. **(A, B)** Two representative NJ tree topologies constructed using aligned amino acid sequences under different substitution models and gap/missing data treatments. *A. baerii* HPX is consistently placed basal to the Wap65-1 and Wap65-2 clades and clusters with holostean (gar; *Lepisosteus oculatus*) HPX, forming a distinct lineage within Actinopterygii. Bootstrap values (1000 replicates) are shown at major nodes. The tree is rooted with three cartilaginous fish (Chondrichthyes) sequences as outgroup taxa. Node numbers (ND1 - ND18 for **A** and ND1 – ND17 for **B**) and their bootstrap value ranges are provided below each tree.

Supplementary Figure S5. Tissue-specific *HPX* mRNA expression normalized using three reference genes. Comparison of HPX transcript levels across 11 tissues from *A. baerii* fingerlings, analyzed by RT-qPCR and normalized independently to *RPL5, RPL7*, or *RPL7A*. Each panel shows relative expression levels using log (left) or linear (right) scaling. Despite variation in fold values, all three normalization strategies produced consistent tissue-specific expression patterns, with the liver showing dominant expression, followed by skin and kidney. Statistical groupings (lowercase letters) indicate significant differences among tissues ($p < 0.05$, ANOVA with Tukey's post hoc test).

Supplementary Figure S6. Survival analysis of *A. baerii* fingerlings following intraperitoneal injection of *A. hydrophila*. Kaplan–Meier survival curves showing percent survival pattern after injecting across a wide range of bacterial concentrations (2×10^4 to 2×10^9 CFU/g body weight), along with a saline-injected control. Mortality occurred rapidly in a dose-dependent manner, with complete mortality at 2×10^9 CFU/g BW. Approximately 50% survival was observed at 2×10^5 CFU/g BW, indicating an approximate LD_{50}, while 2×10^4 CFU/g was considered a sublethal dose. Different letters indicate significant difference among groups based on Kaplan-Meier test at $p < 0.05$ using IBM SPSS statistics tool (ver. 20.02).

Supplementary Figure S7. Validation of *HPX* qPCR expression consistency using conserved-region primers. Relative quantification (RQ) values obtained with different *HPX* primer pairs (primer pairs #1, #2, and #3). Primer pair #1 was designed to amplify the junction between the coding region and the 3′-untranslated region, whereas primer pairs #2 and #3 target regions conserved across publicly available *Acipenser HPX*-like mRNA sequences within the coding region. PCR efficiencies for primer pairs #1–#3 were 1.97, 1.98, and 1.95, respectively. Primer sequences are listed in Supplementary **Table S1. (A, B)** Scatter plots with Deming regression lines (orange) and 1:1 lines (blue) comparing log_{10}-transformed RQ values from primer pair #1 vs primer pair #2 **(A)** and primer pair #1 vs primer pair #3 **(B)**. The equations, Pearson's correlation coefficients (r), and Lin's concordance correlation coefficients (CCC) are shown in each panel. **(C, D)** Bland–Altman plots comparing primer pair #1 with primer pair #2 **(C)** and primer pair #1 with primer pair #3 **(D)**. Solid lines indicate the mean bias, and dotted lines indicate the 95% limits of agreement (LOA). Values are shown both in log_{10} scale and in the corresponding fold-change scale (in parentheses). Details on the objectives, methodology, and statistical evaluation for Supplementary Figure S7 are provided below.

Details on data in Supplementary Figure S7

- **Objective**

The objective was to assess whether the original *HPX* qPCR primer pair (primer pair #1) used in the submitted study accurately and reliably quantified *HPX* mRNA expression without bias from potential sequence variation (e.g., allelic polymorphisms or undetected paralogs). To address this, two additional primer pairs (primer pairs #2 and #3) were designed to target highly conserved regions of the *HPX* coding sequence across multiple Acipenser species. This design strategy aimed to maximize inclusiveness across potential interspecific or allelic variants and

evaluate the agreement of expression measurements from these conserved-region primers relative to the original set.

Methodology

Primer design: Primer pair #1 (original): targeted the junction between the coding sequence (CDS) and the 3′ untranslated region (3′-UTR). Primer pairs #2 and #3 (conserved-region): designed from an alignment of publicly available *Acipenser HPX*-like mRNA sequences (*A. baerii, A. ruthenus, A. oxyrinchus, A. gueldenstaedtii*) to target regions within the CDS showing complete conservation. Primer sequences are listed in Supplementary **Table S1**.

PCR efficiency and specificity: PCR efficiencies (*E*) were determined from standard curves: 1.97 (#1), 1.98 (#2), and 1.95 (#3). Specificity was confirmed by single peaks in melting curves and single expected-size amplicons on agarose gels.

Sample selection: Fifteen representative biological samples were selected from four experimental sets in the main study:

- Tissue distribution — liver (highest expression), skin (second highest), intestine (lowest).
- Ontogeny — hatching stage (0 DPH), 8 DPH (peak), 9 DPH (post-peak drop).
- Bacterial challenge *(A. hydrophila)* — kidney at saline control, 2 × 10^4, 2 × 10^5, and 2 × 10^6 cfu/g BW doses (12 hpi); liver at 0 and 12 hpi (2 × 10^4 cfu/g BW).
- Thermal challenge — skin at 16 °C control, 25 °C+day 0, and 25 °C+day 7.

qPCR and data processing: RNA extraction, cDNA synthesis, and qPCR conditions matched those in the main study. *RPL5* was used as the reference gene for normalization. For each sample and primer pair, mean Ct values from three technical replicates were converted to relative quantities (RQ) accounting for primer efficiency. Log_{10}(RQ) values were used for statistical comparisons.

Statistical analyses: All comparisons used primer pair #1 as the reference. Pearson's correlation coefficient (r) and Lin's concordance correlation coefficient (CCC) for linear association and overall agreement. Deming regression (accounts for error in both axes) to estimate slope and intercept with 95% confidence intervals (CI). Bland–Altman analysis to assess mean bias and 95% limits of agreement (LOA), expressed in both $\log_{10}$ and fold-change scales. Two one-sided tests (TOST) for equivalence, with bounds set at ±20% on the fold-change scale. All computations were performed in Python 3.10 using open-source libraries: pandas (data handling), numpy (numerical computations), scipy.stats (statistical tests), and matplotlib (plotting). Deming regression and Lin's CCC were implemented via custom code following published formulae. Bland–Altman and TOST calculations were performed directly in Python.

Results and interpretation

Primer pair #1 vs primer pair #2 (conserved set): very high correlation (r = 0.99974) and agreement (CCC = 0.99963). Deming regression slope = 0.9970 (95% CI: 0.972 – 1.022), intercept = −0.0077 (95% CI: −0.097 – 0.081). Bland–Altman bias = −0.0168 $\log_{10}$ (fold bias = 0.962×, ~3.8% lower); LOA: 0.854× to 1.084×. TOST confirmed equivalence within ±20% ($p < 0.001$).

Primer pair #1 vs primer pair #3 (conserved set): Very high correlation (r = 0.99896) and agreement (CCC = 0.99891). Deming regression slope = 1.0102 (95% CI: 0.998 – 1.022), intercept = −0.0338 (95% CI: −0.082

– 0.014). Bland–Altman bias = −0.00239 $\log_{10}$ (fold bias = 0.995×, ~0.5% lower); LOA: 0.779× to 1.270×. TOST confirmed equivalence within ±20% ($p < 0.001$) (Supplementary **Table S3**).

Interpretation: Both conserved-region primer sets produced expression measurements that were statistically indistinguishable from those obtained with the original primer set. Minimal bias, narrow LOA, and high CCC values indicate near-perfect agreement, confirming that the original primer pair reliably captured *HPX* transcript abundance across diverse sample types. These results strongly suggest that the observed expression trends in the main study are robust to primer design and unlikely to be confounded by allelic variation or undetected paralogs. These analyses demonstrate high correlation, near-unity slopes, minimal bias, and narrow LOA, supporting the equivalence of results obtained with different primer pairs.
