# Supplementary material for: Molecular Characterization of Hemopexin in the Siberian Sturgeon (Acipenser baerii): Evolutionary Insights and Differential Expression Under Immune and Thermal Stresses
Source: Int J Mol Sci. 2025 Aug 17;26(16):7934. doi: 10.3390/ijms26167934 (PMC12386703; doi:10.3390/ijms26167934)
Supplement: Supplementary file 1 [file ijms-26-07934-s001.zip › Suppl Fig S4A-NJ tree topology-1.pdf]

Suppl. Fig. S4A

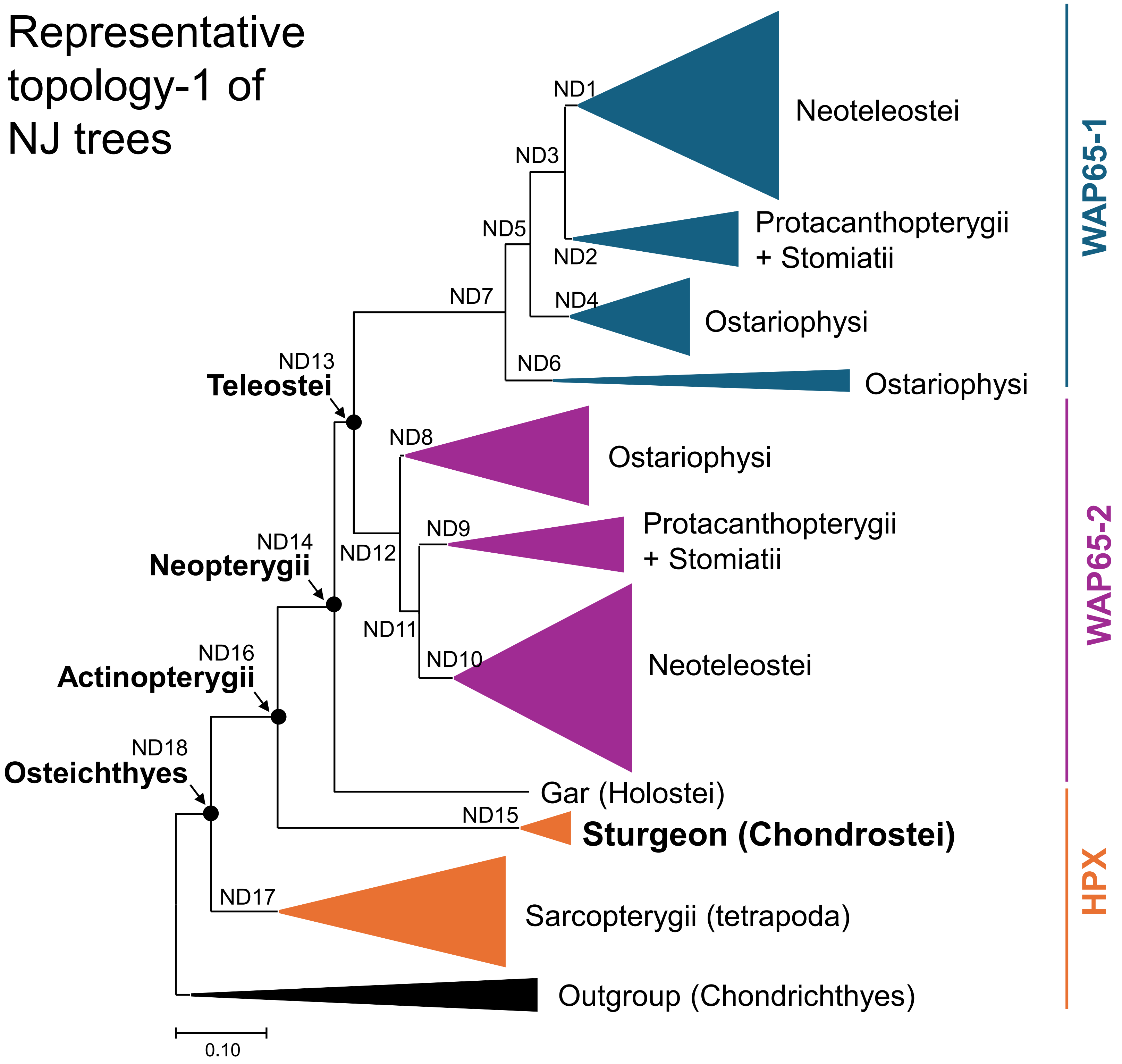

Node #	Bootstrap (%) range	Node #	Bootstrap (%) range
ND1	67–76	ND10	95–100
ND2	54–61	ND11	61–72
ND3	87–98	ND12	96–100
ND4	72–97	ND13	53–91
ND5	56–89	ND14	83–99
ND6	88–95	ND15	99–100
ND7	99–100	ND16	98–100
ND8	42–95	ND17	98–99
ND9	87–96	ND18	90–98
