# Supplementary material for: Molecular Characterization of Hemopexin in the Siberian Sturgeon (Acipenser baerii): Evolutionary Insights and Differential Expression Under Immune and Thermal Stresses
Source: Int J Mol Sci. 2025 Aug 17;26(16):7934. doi: 10.3390/ijms26167934 (PMC12386703; doi:10.3390/ijms26167934)
Supplement: Supplementary file 1 [file ijms-26-07934-s001.zip › Suppl Fig S4B-NJ tree topology-2.pdf]

Suppl. Fig. S4B

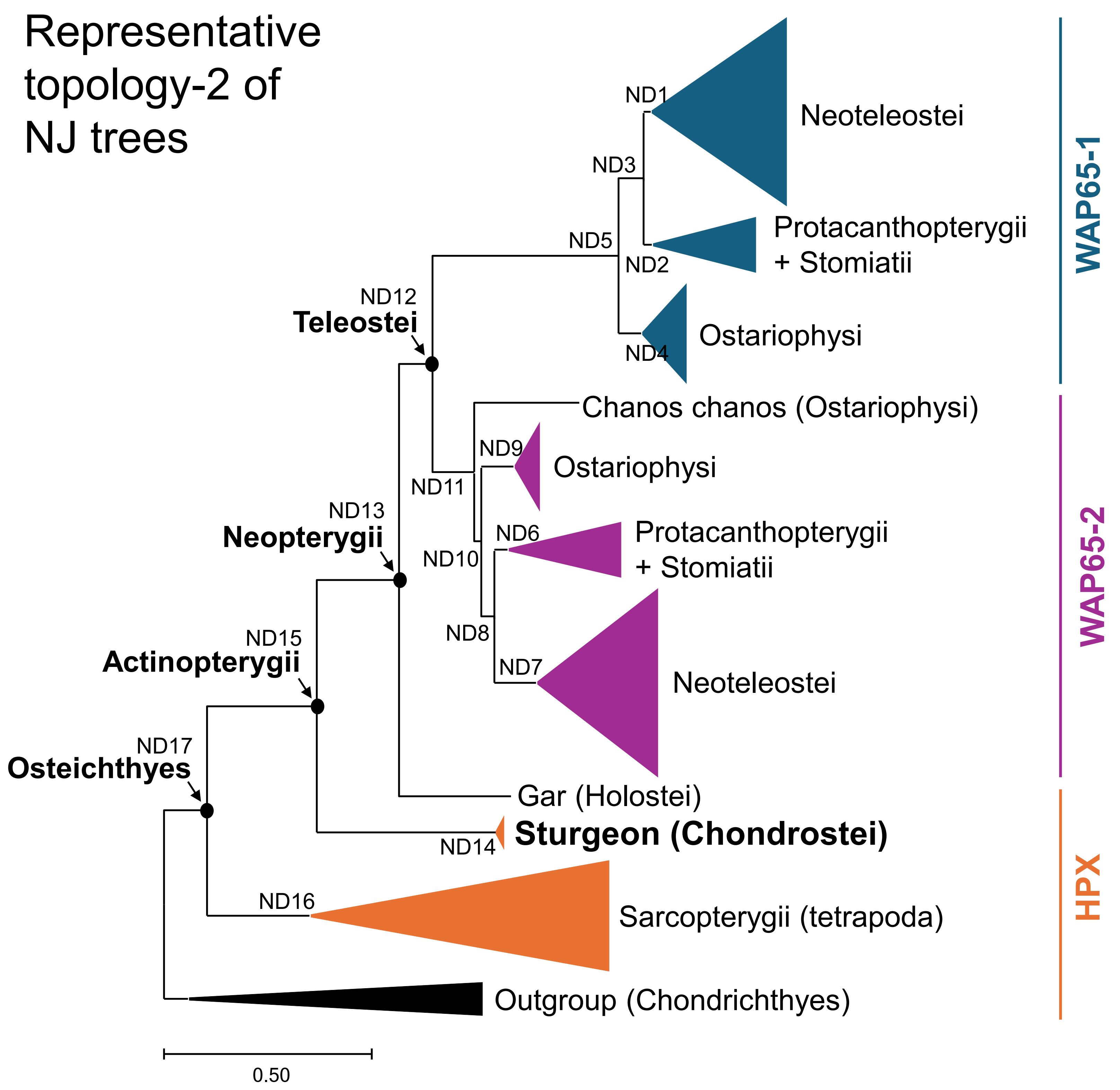

Node #	Bootstrap (%) range	Node #	Bootstrap (%) range
ND1	60–87	ND10	30–43
ND2	52–58	ND11	97–99
ND3	88–93	ND12	90–97
ND4	74–80	ND13	94–99
ND5	99	ND14	99
ND6	79–83	ND15	99
ND7	99	ND16	99
ND8	68–74	ND17	97
ND9	93–98		
