# Supplementary material for: Molecular Characterization of Hemopexin in the Siberian Sturgeon (Acipenser baerii): Evolutionary Insights and Differential Expression Under Immune and Thermal Stresses
Source: Int J Mol Sci. 2025 Aug 17;26(16):7934. doi: 10.3390/ijms26167934 (PMC12386703; doi:10.3390/ijms26167934)
Supplement: Supplementary file 1 [file ijms-26-07934-s001.zip › Suppl Table S1-Primer sequences.pdf]

Supplementary Table S1. Oligonucleotide PCR primers used in this study

Primer name	Sequence (5'-3')	Purpose	Thermal cycling conditions
AB_HPX-FW	TGAGGAATCCATCCCAAGAG	Isolation of full ORF of hemopexin cDNA (amplicon = 1743 bp)	94 ºC for 2 min (initial denaturation) followed by 30 cycles at 94 ºC for 30 s, 58 ºC for 30 s and 72 ºC for 30 s
AB_HPX-RV	GGTACAGAAGTTGTCTGTCC		
AB_HPX-RTq1F	TGTTGACATGTCAGAGTCGC	RT-qPCR assay of hemopexin gene (primer pair #1; amplicon = 258 bp)	94 ºC for 2 min (initial denaturation) followed by 45 cycles at 94 ºC for 20 s, 58 ºC for 20 s and 72 ºC for 20 s
AB_HPX-RTq1R	TATGTGTGCTGCACAGTGCT		
AB_HPX-RTq2F	CAGGGAGATCAGGTGTACAT	RT-qPCR assay of hemopexin gene (primer pair #2; amplicon = 247 bp)	
AB_HPX-RTq2R	CACACATTGCTGCATCCACA		
AB_HPX-RTq3F	CATGAAGTGTCCTGACAGAG	RT-qPCR assay of hemopexin gene (primer pair #3; amplicon = 229 bp)	
AB_HPX-RTq3R	CCAGGTGAAGACAGCATTGA		
AB_TF-RTq1F	CAGAGATGTGGTCGTCAGAT	RT-qPCR assay of transferrin gene (amplicon = 261 bp)	
AB_TF-RTq1R	TGCTGGCACTTGTGGAATGT		
AB_HSP70-RTq1F	GAAGCTGCTGCAGGATTTCT	RT-qPCR assay of heat shock protein 70 gene (amplicon = 256 bp)	
AB_HSP70-RTq1R	AGAGTAGGTGGTGAAGGTCT		
AB_RPL5-RTq1F	TCACAGCACCAAACGTTTCC	Amplification of internal controls for RT-qPCR normalization of target genes (amplicons = 241, 207, and 238 bp, for *RPL5*, *RPL7*, and *RPL7A*, respectively)	
AB_RPL5-RTq1R	TTCATGCACAGGATTCTCCC		
AB_RPL7-RTq1F	GGCAAGAGTGGTATCATCTG		
AB_RPL7-RTq1R	GTTCATCCTCCTGATGAGTC		
AB_RPL7A-RTq1F	CCATACTGCATTGTCAAGGG		
AB_RPL7A-RTq1R	TGGCCTTTGCTTTTTCCAGC		

For primer design, the nucleotide sequences of each gene—except *HPX* (GenBank accession: PV871511) and *TF* (PP145332)—were retrieved from our local *Acipenser baerii* transcriptomic database. For each target gene, multiple matching transcript reads were assembled into a consensus contig. The amplified region was validated by direct sequencing of PCR products, and gene identity was confirmed via BLASTx annotation against the NCBI GenBank database.

Three primer pairs (#1 - #3) were used for *HPX* RT-qPCR expression assay. Primer pair #1 (q1F/q1R) was a study-specific design based on the *A. baerii HPX* sequence. Primer pairs #2 (q2F/q2R) and #3 (q3F/q3R) were designated to the coding regions conserved across all publicly available *Acipenser HPX*-like sequences with the objective of maximizing inclusiveness for potential allelic variants and minimizing the risk of sequence-specific bias.
