# Supplementary material for: Molecular Characterization of Hemopexin in the Siberian Sturgeon (Acipenser baerii): Evolutionary Insights and Differential Expression Under Immune and Thermal Stresses
Source: Int J Mol Sci. 2025 Aug 17;26(16):7934. doi: 10.3390/ijms26167934 (PMC12386703; doi:10.3390/ijms26167934)
Supplement: Supplementary file 1 [file ijms-26-07934-s001.zip › Suppl Table S2-Taxon sampling.pdf]

Supplementary Table S2. Summarized information on amino acid sequences used for multiple sequence alignments and phylogenetic analysis in this study

Species (common name)	Taxonomic position		Type*	GenBank accession code	No. AA residues	Mw (Da)	pI
Ginglymostoma cirratum (nurse shark)	Chondrichthyes	Elasmobranchii	HPX	ADU15819.1	442	51162.54	6.46
Leucoraja erinacea (little skate)		Elasmobranchii	HPX	ADU15818.1	437	48714.09	6.22
Callorhinchus milii (elephant shark)		Holocephali	HPX	AFP00948.1	482	54751.24	7.91
Sus scrofa (pig)	Sarcopterygii (tetrapod)	Mammalia	HPX	NP_999118.1	459	51305.70	6.59
Bos taurus (cattle)		Mammalia	HPX	AAI02688.1	459	52209.28	7.90
*Homo sapiens (*human)		Mammalia	HPX	AAA58678.1	462	51676.37	6.55
Mus musculus (house mouse)		Mammalia	HPX	AAH19901.1	460	51340.78	7.92
Rattus norvegicus (Norway rat)		Mammalia	HPX	AAA41337.1	460	51290.85	7.58
Gallus gallus (chicken)		Sauropsida-Aves	HPX	XP_015136422.1	450	49672.37	5.24
Coturnix japonica (Japanese quail)		Sauropsida-Aves	HPX	XP_015708049.1	449	49715.78	5.80
Pseudonaja textilis (Eastern brown snake)		Sauropsida-Snake	HPX	XP_026577418.1	471	52234.81	6.57
Notechis scutatus (mainland tiger snake)		Sauropsida-Snake	HPX	XP_026544192.1	490	54033.91	6.61
Alligator mississippiensis (American alligator)		Sauropsida-Crocodile	HPX	KYO35827.1	460	50623.57	6.33
Acipenser baerii (Siberian sturgeon)	Actinopterygii	Chondrostei	HPX	This study	455	52833.40	6.07
Acipenser ruthenus (sterlet)		Chondrostei	HPX	RXN00866.1	484	56536.29	6.00
Acipenser ruthenus (sterlet)		Chondrostei	HPX	XP_033866453.2	455	52702.13	6.01
Lepisosteus oculatus (spotted gar)	Neopterygii	Holostei	HPX	XP_015219463.1	476	54403.70	5.82
Chanos chanos (milkfish)	Teleostei	Ostariophysi>Gonorynchiformes	Wap65-1	XP_030641828.1	428	48858.66	5.73
			Wap65-2	XP_030636491.1	444	50506.10	6.16
Danio rerio (zebrafish)		Ostariophysi>Cypriniformes	Wap65-1	AAI55108.1	447	50998.67	6.18
			Wap65-2	XP_005173505.1	435	49383.81	5.81
Cyprinus carpio (common carp)		Ostariophysi>Cypriniformes	Wap65-1	BAB60809.1	439	50045.31	5.58
			Wap65-2	ATP66527.1	445	50502.89	5.71
Carassius auratus (goldfish)		Ostariophysi>Cypriniformes	Wap65-1	BAA08928.1	445	50769.14	5.62
			Wap65-2	XP_026060067.1	444	50497.14	5.71
Carassius carassius (crucian carp)		Ostariophysi>Cypriniformes	Wap65-1	BAP90357.1	445	50806.25	5.71
			Wap65-2	XP_059410071.1	447	51011.79	5.84
Misgurnus mizolepis (mud loach)		Ostariophysi>Cypriniformes	Wap65-1	AEM60430.1	457	51770.59	6.52
			Wap65-2	AEM60431.1	446	50714.28	5.69
Paramisgurnus dabryanus (large scale loach)		Ostariophysi>Cypriniformes	Wap65-1	XP_065107897.1	457	51769.60	6.58
			Wap65-2	ALM01492.1	446	50801.36	5.64
Pygocentrus nattereri (red-bellied piranha)		Ostariophysi>Characiformes	Wap65-1	XP_017540730.1	495	56050.23	5.87
			Wap65-2	XP_017565513.1	440	50013.00	6.41
Ictalurus punctatus (channel catfish)		Ostariophysi>Siluriformes	Wap65-1	ABW07851.1	478	54330.00	5.52
			Wap65-2	ABW07852.1	443	50602.35	6.14

Esox lucius (northern pike)		Protacanthopterygii>Esociformes	Wap65-1	XP_010901644.4	513	58165.59	6.06
			Wap65-2	XP_010884702.1	443	49840.28	5.82
Oncorhynchus mykiss (rainbow trout)		Protacanthopterygii>Salmoniformes	Wap65-1	XP_021453746.1	445	50369.65	5.69
			Wap65-2	XP_021464322.1	442	49941.06	5.69
Salmo salar (Atlantic salmon)		Protacanthopterygii>Salmoniformes	Wap65-1	XP_014030085.1	448	50949.39	5.80
			Wap65-2	XP_014007262.1	440	49539.91	5.93
Salmo trutta (river trout)		Protacanthopterygii>Salmoniformes	Wap65-1	XP_029568246.1	457	51853.25	5.78
			Wap65-2	XP_029597560.1	444	49997.39	5.81
Plecoglossus altivelis (ayu)		Stomiatii>Osmeriformes	Wap65-1	CCG97808.1	442	50473.67	5.48
			Wap65-2	CAQ53699.1	439	49757.11	5.85
Scomberomorus niphonius (Japanese Spanish mackerel)	Neoteleostei	Pelagiaria>Scombriformes	Wap65-1	AMW64453.1	426	48742.97	5.38
			Wap65-2	AMW64454.1	436	48489.46	5.52
Hippocampus comes (tiger tail seahorse)		Syngantharia>Syngnathiformes	Wap65-1	XP_019748984.1	435	48568.76	5.30
			Wap65-2	XP_019716296.1	427	49109.14	5.32
Scophthalmus maximus (turbot)		Carangaria>Pleuronectiformes	Wap65-1	AID59463.1	429	48913.76	5.38
			Wap65-2	AID59464.1	436	48856.12	5.22
Paralichthys olivaceus (Japanese flounder)		Carangaria>Pleuronectiformes	Wap65-1	AGT28469.1	434	50138.13	5.59
			Wap65-2	AGT28470.1	433	48869.35	5.29
Seriola dumerili (greater amberjack)		Carangaria>Carangiformes	Wap65-1	XP_022596039.1	426	48902.02	5.40
			Wap65-2	XP_022602175.1	436	48657.82	5.67
Seriola lalandi dorsalis (California yellowtail)		Carangaria>Carangiformes	Wap65-1	XP_023260243.1	426	48839.91	5.39
			Wap65-2	XP_023251217.1	436	48779.19	5.76
Fundulus heteroclitus (mummichog)		Ovalentaria>Cyprinodontiformes	Wap65-1	JAR72799.1	429	49256.32	5.63
			Wap65-2	XP_012731313.1	427	47817.10	5.29
Xiphophorus maculatus (southern platyfish)		Ovalentaria>Cyprinodontiformes	Wap65-1	XP_014325704.1	456	52344.08	6.56
			Wap65-2	XP_005813153.1	430	47744.73	5.33
Oryzias latipes (Japanese medaka)		Ovalentaria>Beloniformes	Wap65-1	BAB97303.1	430	49847.42	6.42
			Wap65-2	BAB97304.1	427	48000.21	5.40
Oryzias melastigma (Indian ricefish)		Ovalentaria>Beloniformes	Wap65-1	XP_024142285.1	471	54877.72	6.28
			Wap65-2	XP_024150328.1	427	47976.26	5.35
Maylandia zebra (zebra mbuna)		Ovalentaria>Cichliformes	Wap65-1	XP_024661279.1	431	49342.26	5.63
			Wap65-2	XP_004573323.2	435	49217.59	5.48
Dicentrarchus labrax (European seabass)		Eupercaria incertae sedis	Wap65-1	ABL75414.1	427	49148.18	5.41
			Wap65-2	DAA12504.1	432	48665.98	5.47
Oplegnathus fasciatus (barred knifejaw)		Eupercaria>Centrarchiformes	Wap65-1	AFE88227.1	431	49341.47	5.79
			Wap65-2	AFE88228.1	434	48676.91	5.35
Lateolabrax japonicus (Japanese sea bass)		Eupercaria>Pempheriformes	Wap65-1	CCA29189.1	429	49280.22	5.27
			Wap65-2	CCA29190.1	436	48726.77	5.22

Sparus aurata (gilthead seabream)		Eupercaria>Spariformes	Wap65-1	ACN54269.1	425	49158.12	5.41
			Wap65-2	XP_030266261.1	435	48533.76	5.50
Tetraodon nigroviridis (spotted green pufferfish)		Eupercaria>Tetraodontiformes	Wap65-1	H3BYZ3	424	48662.82	5.48
			Wap65-2	H3D6Y8	442	49590.46	5.64
Takifugu rubripes (fugu rubripes)		Eupercaria>Tetraodontiformes	Wap65-1	BAD18109.1	425	48728.90	5.66
			Wap65-2	BAD18110.1	442	49470.26	5.46

*Classification of each sequence as *HPX*, *Wap65-1*, or *Wap65-2* was determined based on the phylogenetic analyses conducted in this study.
