# Supplementary figures and images for: Molecular Characterization of Hemopexin in the Siberian Sturgeon (Acipenser baerii): Evolutionary Insights and Differential Expression Under Immune and Thermal Stresses
Source: Int J Mol Sci. 2025 Aug 17;26(16):7934. doi: 10.3390/ijms26167934 (PMC12386703; doi:10.3390/ijms26167934)

Suppl. Fig. S1

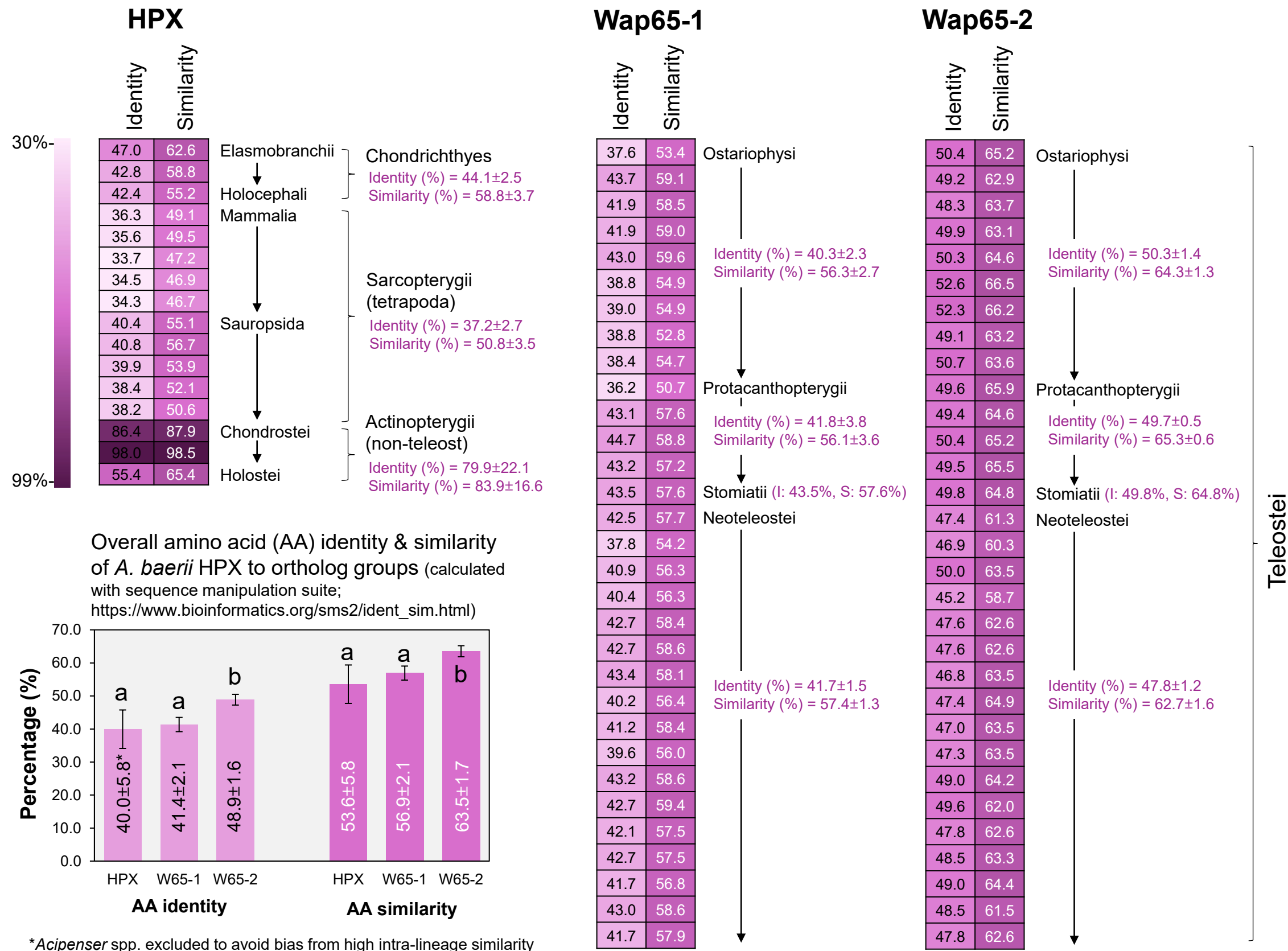

Supplement: Supplementary file 1 [file ijms-26-07934-s001.zip › Suppl Fig S1-Seuqnce Identity.pdf]

Suppl. Fig. S2

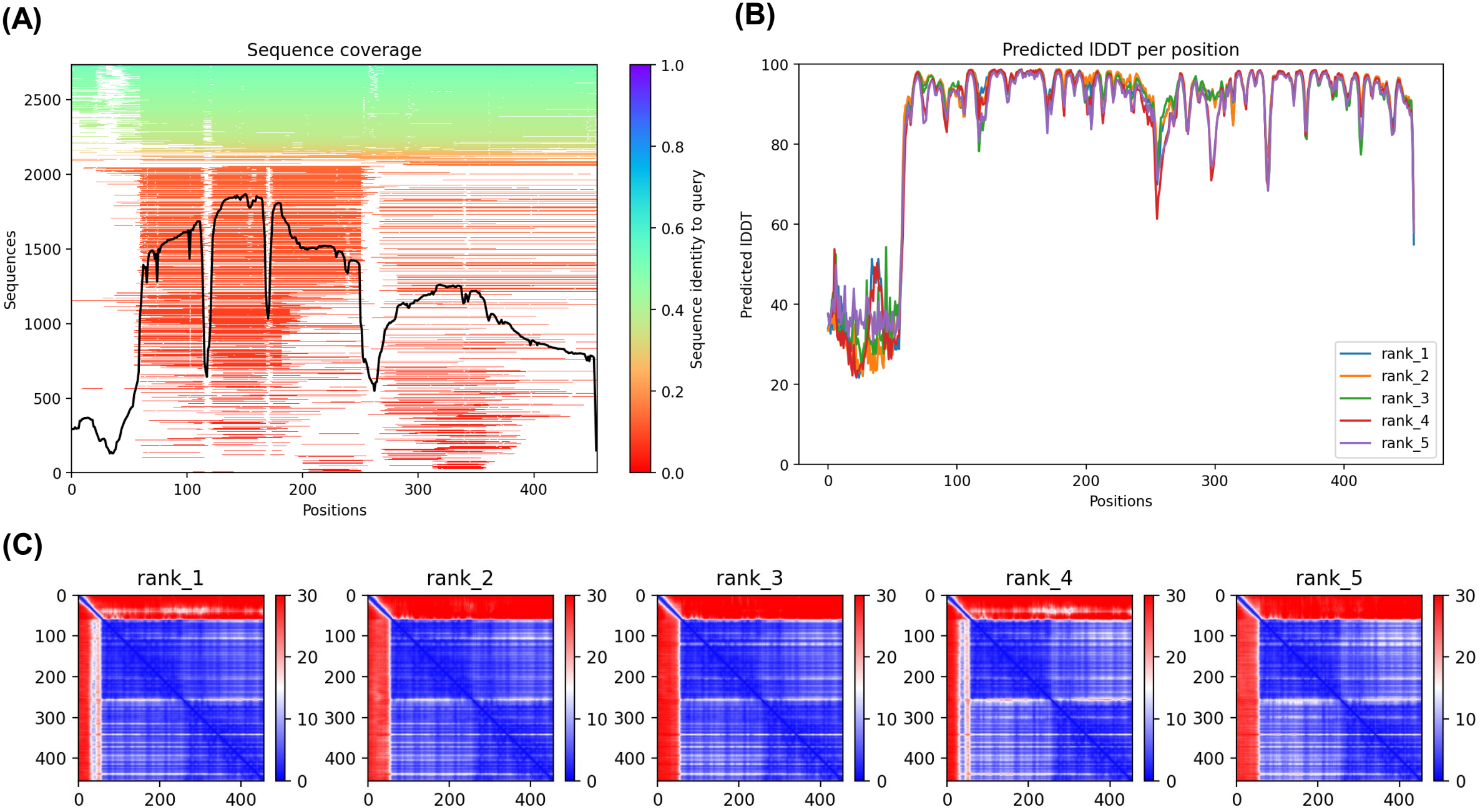

Supplement: Supplementary file 1 [file ijms-26-07934-s001.zip › Suppl Fig S2-Structural modeling quality assessement.pdf]

Suppl. Fig. S3A

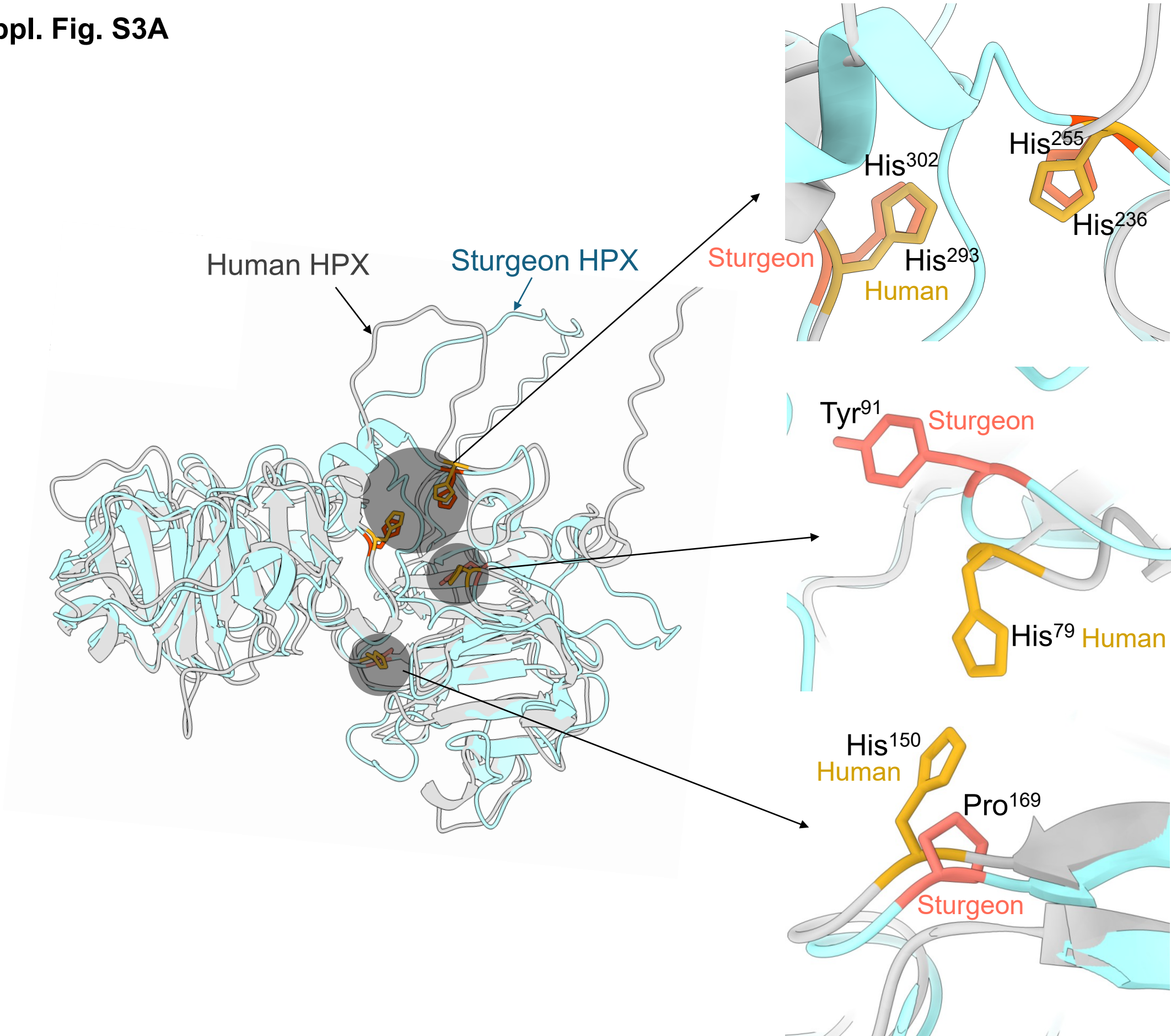

Supplement: Supplementary file 1 [file ijms-26-07934-s001.zip › Suppl Fig S3A-Superimposed (heme binding sites).pdf]

Suppl. Fig. S3B

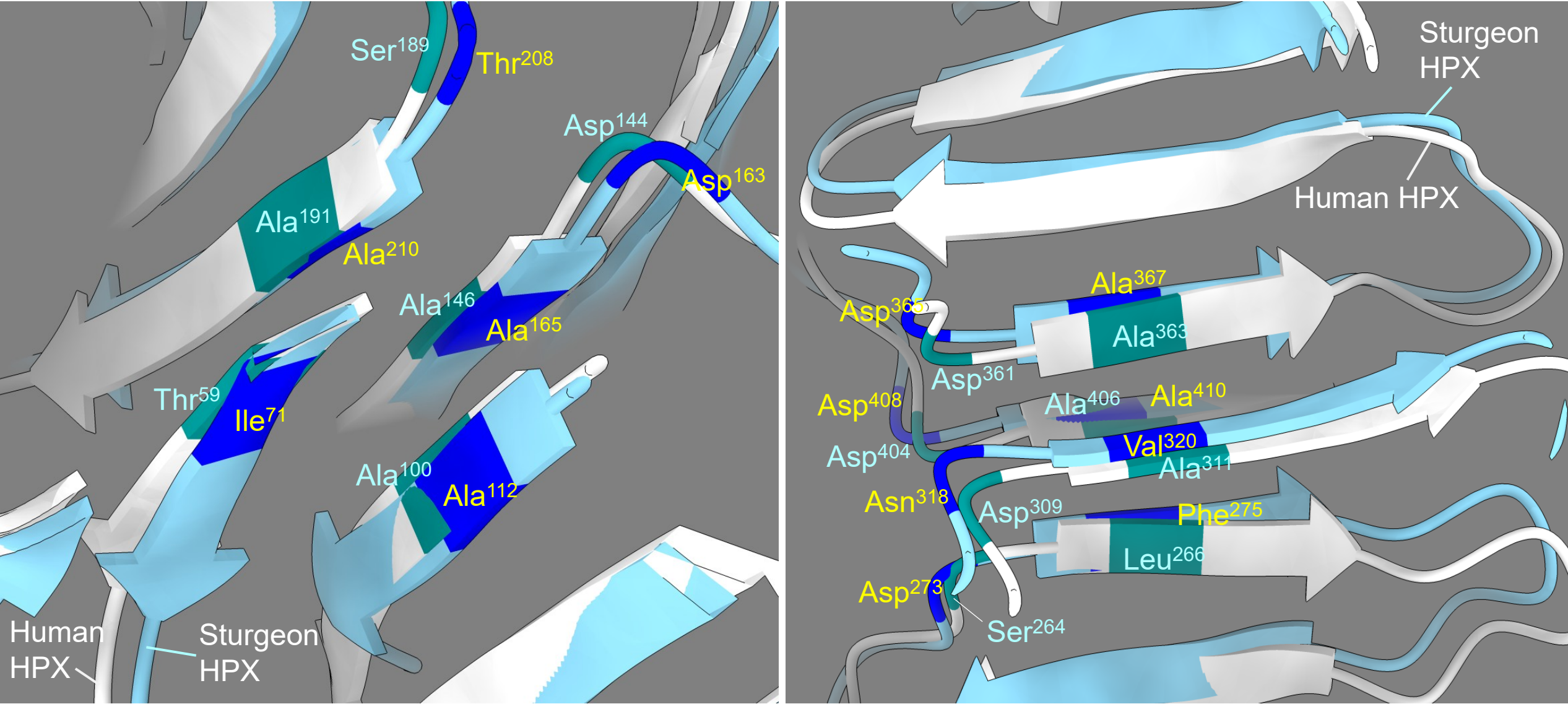

Supplement: Supplementary file 1 [file ijms-26-07934-s001.zip › Suppl Fig S3B-Superimposed (metal binding sites).pdf]

Suppl. Fig. S3C

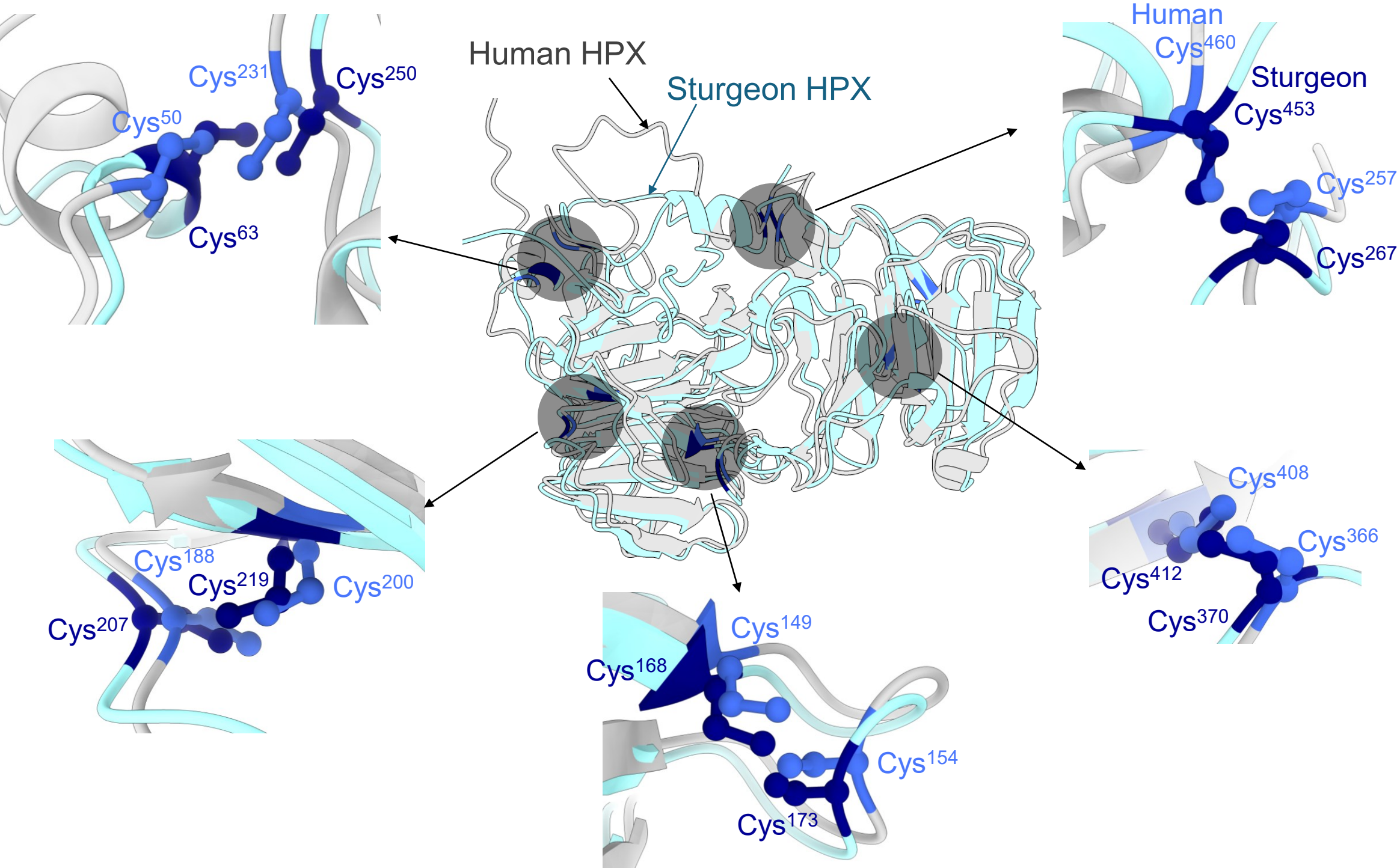

Supplement: Supplementary file 1 [file ijms-26-07934-s001.zip › Suppl Fig S3C-Superimposed (Cys for disulfide bonds).pdf]

Suppl. Fig. S3D

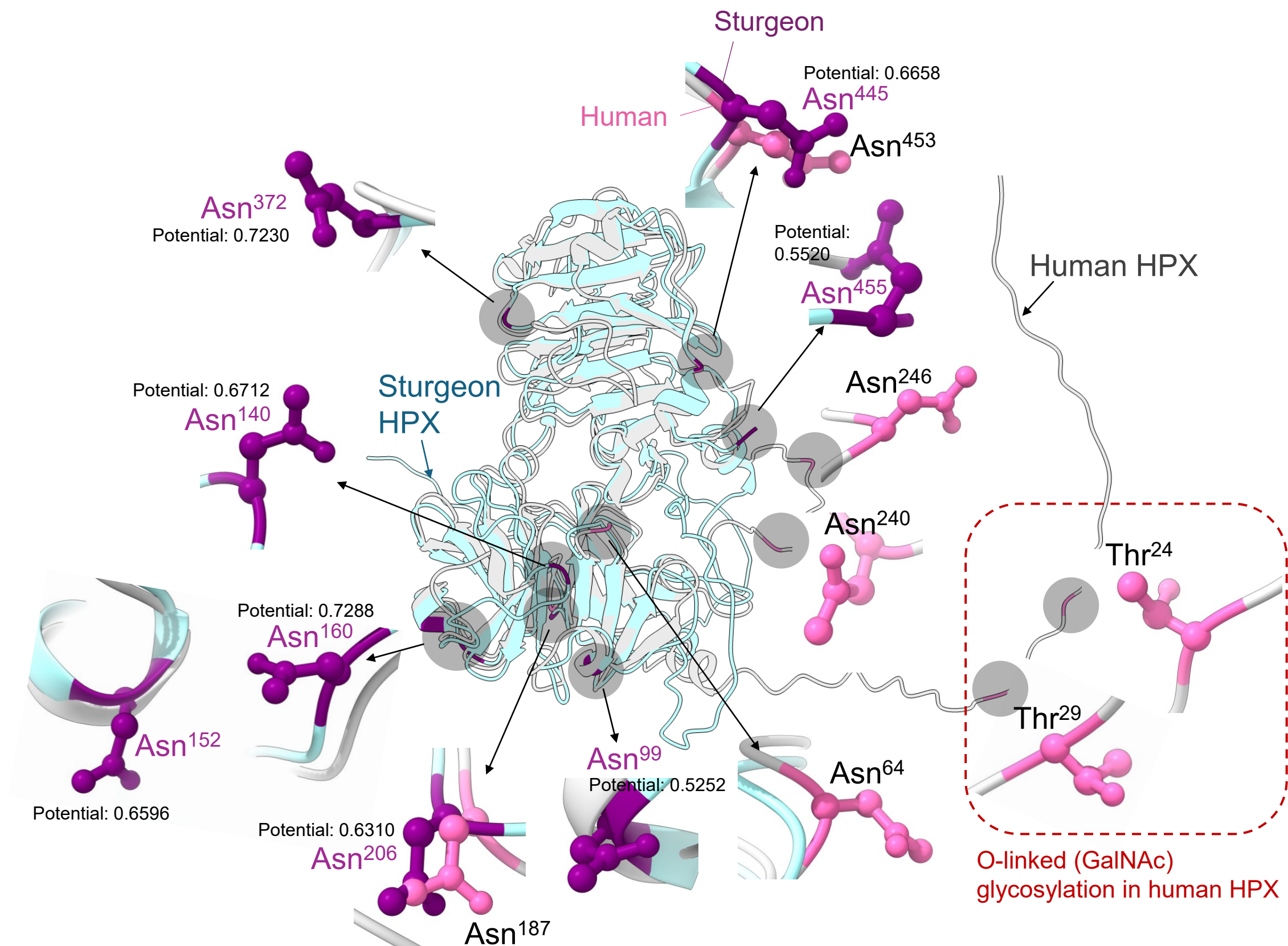

Supplement: Supplementary file 1 [file ijms-26-07934-s001.zip › Suppl Fig S3D-Superimposed (glycosylation sites).pdf]

Suppl. Fig. S5

Normalized based  
on *RPL5*

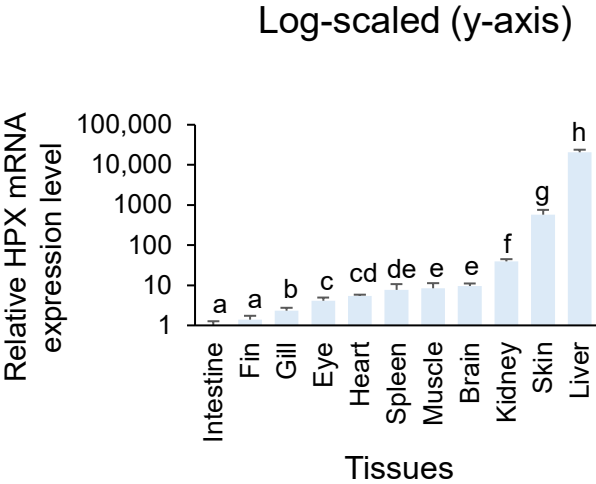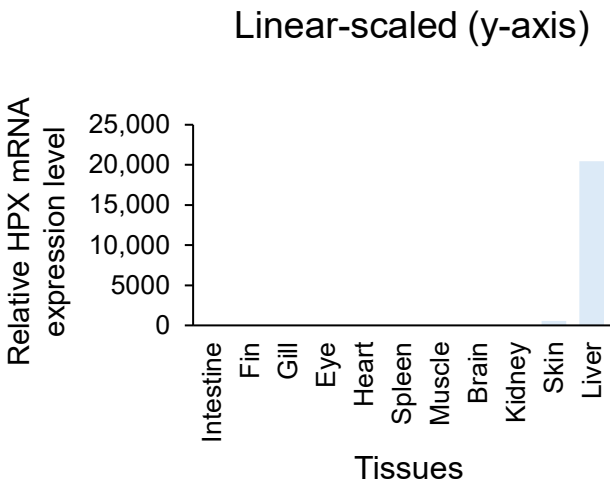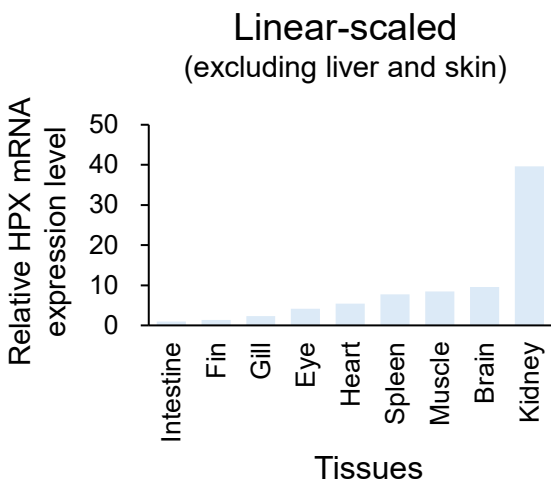

Normalized based  
on *RPL7*

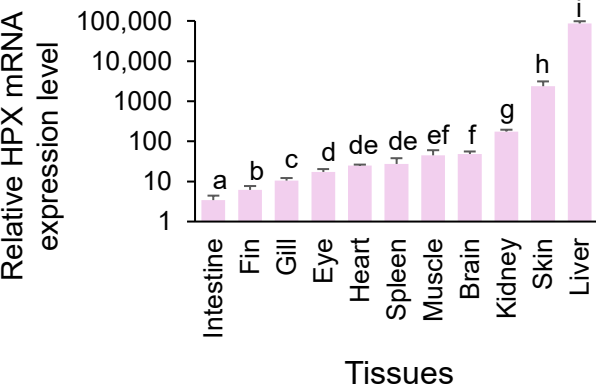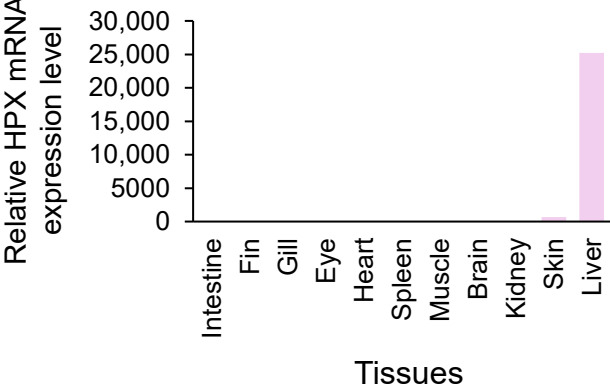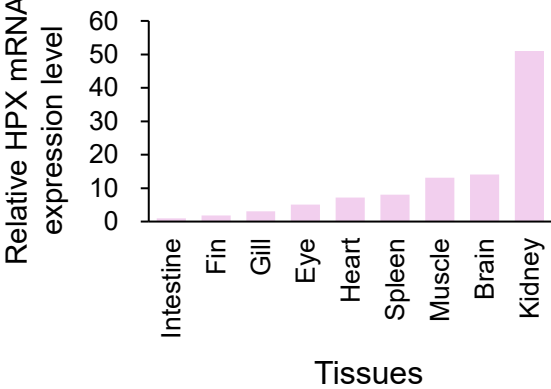

Normalized based  
on *RPL7A*

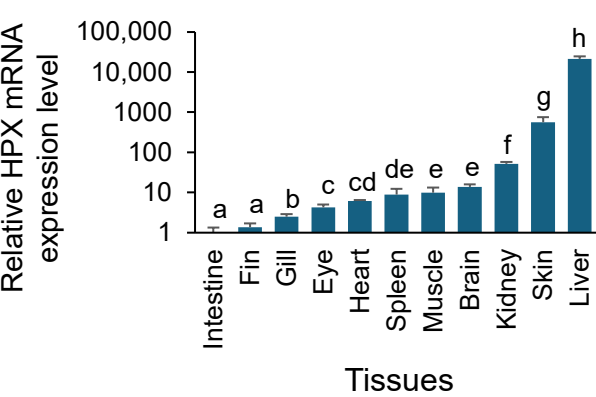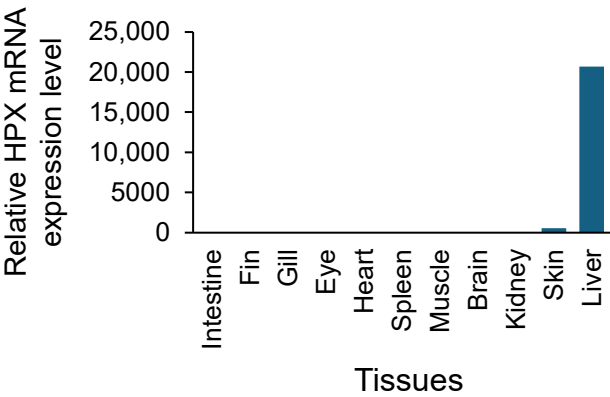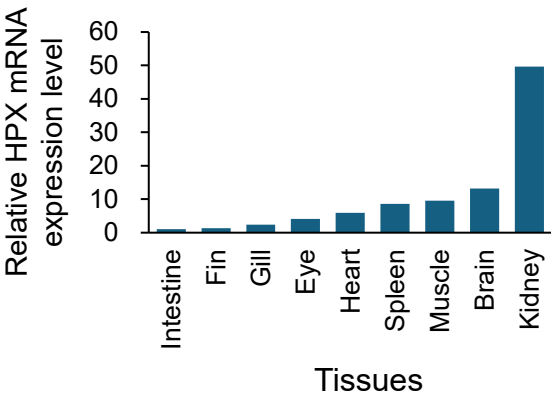

Supplement: Supplementary file 1 [file ijms-26-07934-s001.zip › Suppl Fig S5-Tissue expression normalized control.pdf]

Suppl. Fig. S6

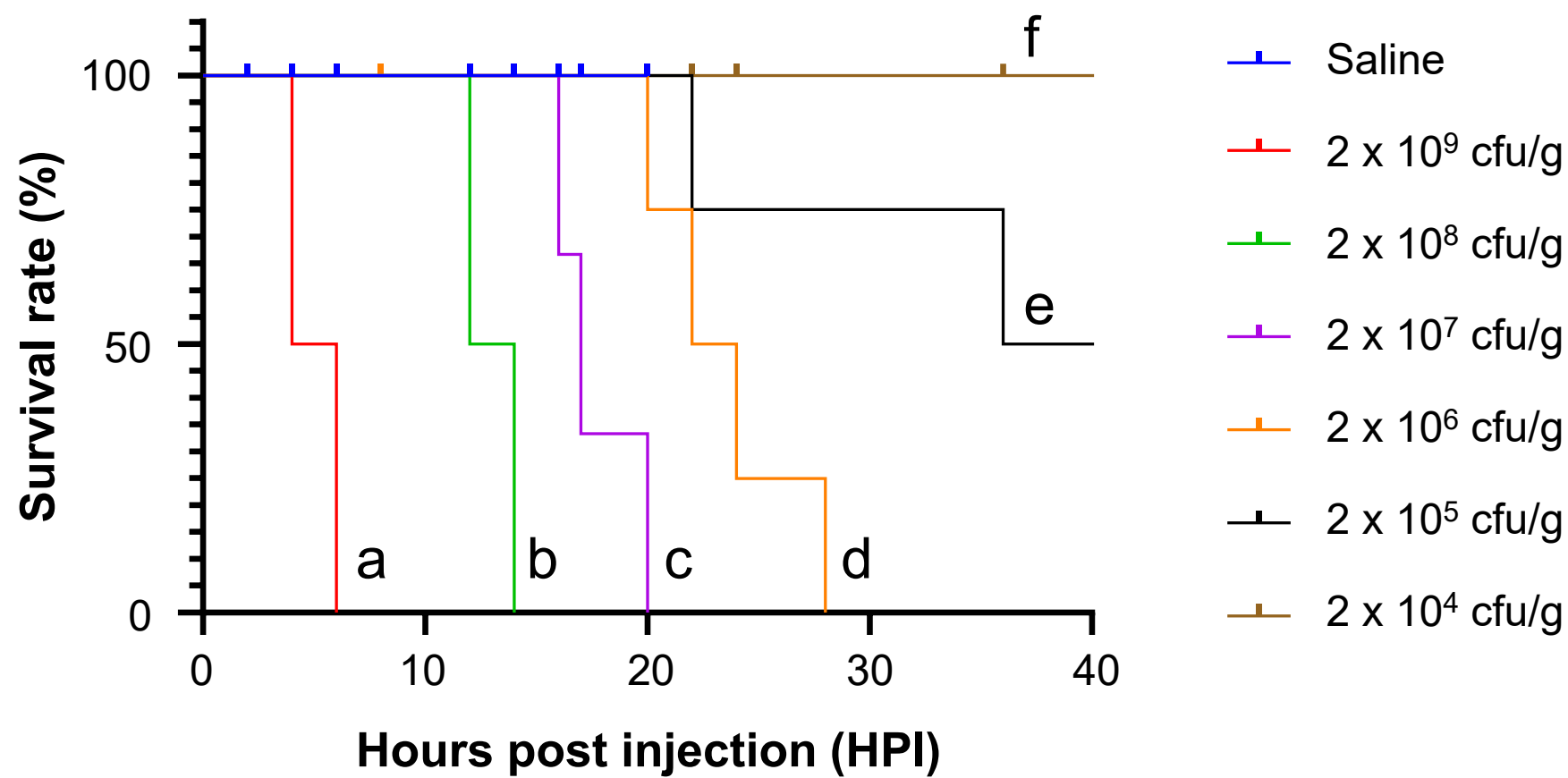

Supplement: Supplementary file 1 [file ijms-26-07934-s001.zip › Suppl Fig S6-Mortality during bacterial challenge.pdf]

Suppl. Fig. S7

(A)

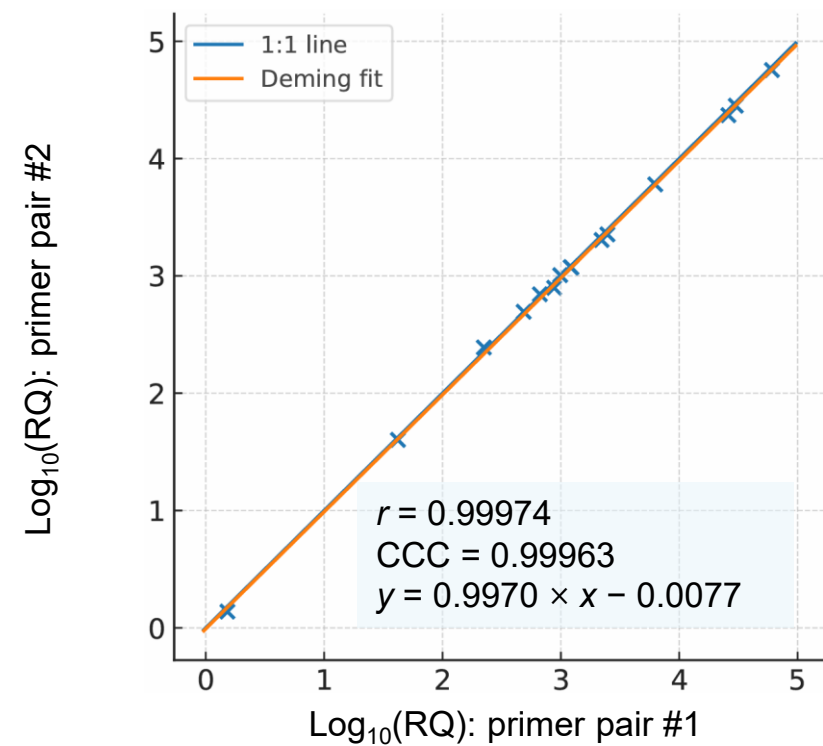

(B)

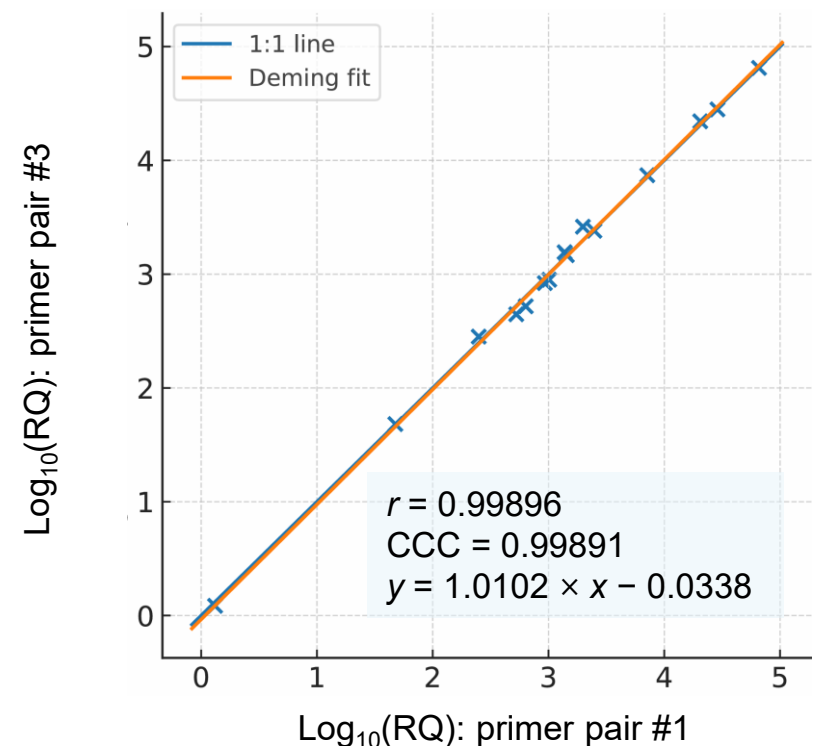

(C)

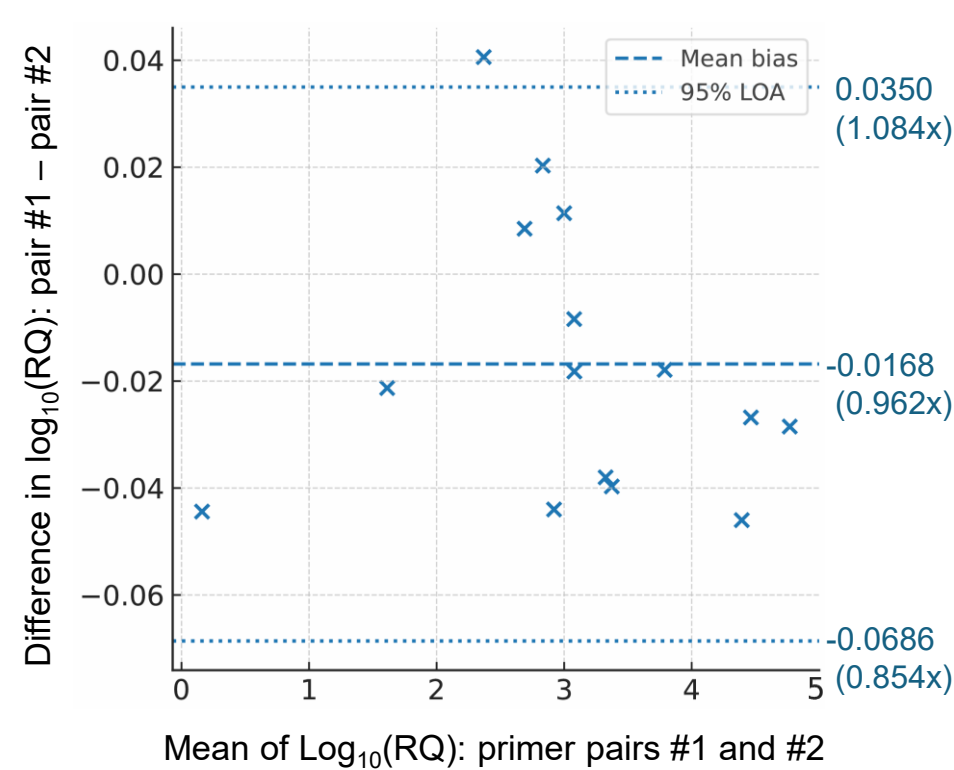

(D)

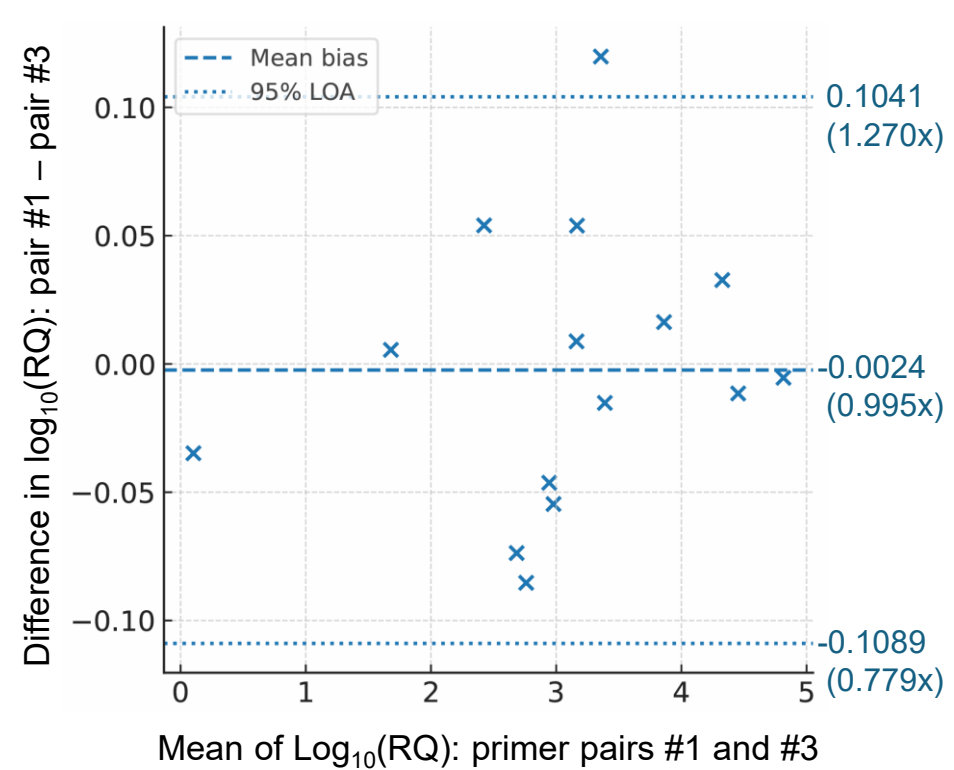

Supplement: Supplementary file 1 [file ijms-26-07934-s001.zip › Suppl Fig S7-Validation of primer pairs for RT-qPCR.pdf]
